# Topical adjunctive treatment with flagellin augments pulmonary neutrophil responses and reduces bacterial dissemination in multidrug-resistant *K. pneumoniae* infection

**DOI:** 10.3389/fimmu.2024.1450486

**Published:** 2024-09-04

**Authors:** Christine C. A. van Linge, Robert F.J. Kullberg, Osoul Chouchane, Joris J. T. H. Roelofs, Wil H. F. Goessens, Cornelis van ‘t Veer, Jean-Claude Sirard, Alex F. de Vos, Tom van der Poll

**Affiliations:** ^1^ Center for Experimental and Molecular Medicine, Amsterdam University Medical Center, Academic Medical Center, University of Amsterdam, Amsterdam, Netherlands; ^2^ Amsterdam Infection & Immunity Institute, Amsterdam, Netherlands; ^3^ Department of Pathology, Amsterdam University Medical Center, Academic Medical Center, University of Amsterdam, Amsterdam, Netherlands; ^4^ Department of Medical Microbiology and Infectious Diseases, Erasmus University Medical Center, Rotterdam, Netherlands; ^5^ Center for Infection and Immunity of Lille, Institut Pasteur de Lille, U1019 - UMR9017, centre hospitalier universitaire (CHU) Lille, Centre national de la recherche scientifique (CNRS), L’institut national de la santé et de la recherche médicale (INSERM), University of Lille, Lille, France; ^6^ Division of Infectious Diseases, Amsterdam University Medical Center, University of Amsterdam, Amsterdam, Netherlands

**Keywords:** pneumonia, *Klebsiella pneumoniae*, antimicrobial resistance, flagellin, respiratory infection

## Abstract

**Objective:**

Antimicrobial resistance is an emerging problem and multi-drug resistant (MDR) *Klebsiella pneumoniae* (*K. pneumoniae*) represents an enormous risk of failing therapy in hospital-acquired pneumonia. The current study aimed to determine the immunomodulatory effect of topical flagellin in addition to antibiotic treatment during respiratory infection evoked by hypervirulent antibiotic-susceptible and antibiotic-resistant *K. pneumoniae* in mice.

**Methods:**

C57BL6 mice were inoculated intranasally with hypervirulent *K. pneumoniae* (K2:O1) which was either antibiotic-susceptible or multi-drug resistant. Six hours after infection, mice were treated with antibiotics intraperitoneally and flagellin or vehicle intranasally. Mice were sacrificed 24 hours after infection. Samples were analyzed for bacterial loads and for inflammatory and coagulation markers.

**Results:**

Flagellin therapy induced neutrophil influx in the lung during antibiotic-treated pneumonia evoked by either antibiotic-susceptible or -resistant *K. pneumoniae*. The pulmonary neutrophil response was matched by elevated levels of neutrophil-attracting chemokines, neutrophil degranulation products, and local coagulation activation. The combined therapy of effective antibiotics and flagellin did not impact *K. pneumoniae* outgrowth in the lung, but decreased bacterial counts in distant organs. Neutrophil depletion abrogated the flagellin-mediated effect on bacterial dissemination and local coagulation responses.

**Conclusion:**

Topical flagellin administration as an adjunctive to antibiotic treatment augments neutrophil responses during pneumonia evoked by MDR-*K. pneumoniae*, thereby reducing bacterial dissemination to distant organs.

## 1 Introduction

Bacterial antimicrobial resistance is an emerging public health threat. In 2019, an estimated 1.27 million deaths were directly attributable to antimicrobial resistance (1). *Klebsiella pneumoniae* (*K. pneumoniae*), belonging to the family of *Enterobacterales*, was one of the six pathogens mostly attributing to these deaths and responsible for more than 250,000 deaths associated with antimicrobial resistance (1). *K. pneumoniae* is one of the most frequent pathogens causing hospital-acquired pneumonia (2), and - in certain areas of the world – an emerging cause of community-acquired pneumonia (3). Pneumonia is associated with a tremendous disease burden: in 2019 lower respiratory tract infections affected 489 million people worldwide, resulting in >2.49 million deaths (4). Moreover, pneumonia is the most common cause of sepsis (5). This, together with the fact that multi-drug resistant (MDR) rates of *K. pneumoniae* are trending upwards, explains that the WHO recognizes this bacterium as a critical global health threat (6) and has taken up *K. pneumoniae* in a list of MDR bacteria most in need of development of alternative therapies.

There is an increasing interest in drugs that can ‘boost’ innate immunity as adjunctive therapy in difficult-to-treat infections (7, 8). Flagellin, the structural protein of bacterial flagella and a known Toll-like receptor 5 (TLR5) ligand, triggers a signaling cascade that results in an inflammatory response (9). Respiratory epithelial cells exposed to flagellin, through TLR5-mediated activation, secrete chemokines, leading to the attraction of immune cells. Recently, it was found that combined treatment with antibiotics and flagellin reduced the bacterial burden and increased survival during respiratory infection with antibiotic-susceptible and antibiotic-resistant *Streptococcus pneumoniae* (*S.* pneumoniae) in mice (10). *S. pneumoniae* is a Gram-positive diplococcus and the most common causative agent of community-acquired pneumonia, unlike *K. pneumoniae*, which is gram-negative and mainly affects hospitalized patients (2). In the context of hospital-acquired infections, it is especially important to study alternative or additional treatments, because antibiotic resistance is more frequent, and patients can be immunocompromised due to underlying illnesses or treatment thereof (8). We here sought to determine the effect of topical flagellin treatment, as an adjunctive to antibiotics, on the host response during pneumonia caused by hypervirulent antibiotic susceptible- and MDR-*K. pneumoniae*. We show that intrapulmonary flagellin instillation in combination with effective antibiotic treatment increased neutrophil influx and neutrophil activation in the lung, and, along with an increase of coagulation responses, reduced bacterial dissemination from the lungs.

## 2 Materials and methods

### 2.1 Mice

C57BL/6 mice were obtained from Charles River (Netherlands) and kept under standard care and specific pathogen-free conditions. All mice were female and at the age of 8-12 weeks during the experiments. Experiments were performed in accordance with the Dutch Experiment on Animals Act and European Directives and approved by the Central Authority for Animal Experiments and the Animal Welfare Body of the Amsterdam University Medical Center (approval numbers DIX17-4125-1-90 and DIX22-16434-1-04).

### 2.2 Bacterial strains

*K. pneumoniae* American Type Culture Collection 43816 (K2:O1) was used in all experiments. The wild-type strain (referred to in this study as *Kpneu*) was confirmed to be susceptible to ceftriaxone *in vivo* (**Supplementary Figure S1**). An isogenic carbapenem-resistant variant of *K. pneumoniae* 43816 (KPC EMC2014, generated via bacterial conjugation (11), referred to in this study as MDR-*Kpneu*) was susceptible to colistin (MIC < 0.5 mg/L), but not to ceftriaxone (MIC > 32 mg/L), and this was confirmed *in vivo* (**Supplementary Figure S1**).

### 2.3 Pneumonia model

Pneumonia was induced as previously described by our group (12). Briefly, mice were inoculated intranasally (i.n.) with approximately 1x10^4^ colony-forming units (CFUs) of *Kpneu* or MDR-*Kpneu*. Mice were sacrificed 24 hours after infection at which point lung, bronchoalveolar lavage fluid (BALF), bronchial epithelial brushes, spleen, and blood were harvested as described (12).

### 2.4 Treatment of mice

At 6 hours after bacterial inoculation, mice were treated intraperitoneally (i.p.) with either 20 mg/kg ceftriaxone (Hikma) (13, 14) or 40 mg/kg colistin (Teva) (15). Simultaneously, mice were treated i.n. with 10 μg flagellin, or a similar volume (30 μl) of vehicle (phosphate buffered saline). Recombinant flagellin originated from the *Salmonella enterica* serovar Typhimurium ATCC14028 flagellin FliC (accession number P06179) and was produced as described (16) (Statens Serum Institut). This dose was based on earlier studies demonstrating the immunostimulatory capacity of i.n. administered flagellin (10). For fibrinogen depletion, mice were treated i.p. with 3 IU ancrod (National Institute for Biological Standards and Control) or vehicle control (saline) 24 hours, and immediately before bacterial infection, as described (17). For antibody-mediated neutrophil depletion, mice were treated i.p. with 50 μg anti-Ly6G antibody (clone 1A8) or isotype control (rat IgG2a,κ, clone 2A3; both from Bio X Cell) 48, 24, and 3 hours prior to bacterial inoculation. In addition, anti-Ly6G and isotype control-treated mice received 100 μg anti-rat κ immunoglobulin light chain antibody (clone MAR18.5; Bio X Cell) as a secondary antibody 46 and 1 hour prior to bacterial inoculation, as described (18).

### 2.5 Determination of bacterial numbers

Lungs and spleen were homogenized and serial dilutions of the lysates, and blood were plated on blood-agar plates for determination of CFUs as previously described (12).

### 2.6 Cell analysis by flow cytometry

Different cell populations in BALF were determined by flow cytometry (19). Briefly, BALF cells were resuspended in FACS buffer (5% BSA, 0.35 mM EDTA, 0.01% NaN3). Cell staining was performed according to the manufacturer’s recommendations using Fixable Viability Dye eFluor780 (Thermo Fisher), rat anti-mouse-CD45 PE-eFluor610 (30-F11), rat anti-mouse Siglec-F AlexaFluor647 (clone E50-2440); and rat anti-mouse Ly-6G FITC (clone 1A8; all Biolegend). Flow cytometry was performed using a Cytoflex-S (Beckman Coulter) and data were analyzed using FlowJo software (Becton Dickinson). The gating strategy is shown in **Supplementary Figure S2**. Neutrophils were defined as CD45^+^Ly6G^+^SiglecF^-^. Neutrophil numbers in BALF were established using Precision Count Beads (BD Bioscience).

### 2.7 Analysis of BALF, plasma, and lung homogenates

Chemokine (C-X-C motif) ligand 1 and 5 (CXCL1, CXCL5), chemokine (C-C motif) ligand 20 (CCL20), myeloperoxidase (MPO), elastase, and IgM were determined in BALF by ELISA, according to the manufacturer’s instructions (R&D Systems). Tumor necrosis factor (TNF) and interleukin 6 (IL-6) were quantified in BALF and citrated plasma by Cytometric Bead Array, according to the manufacturer’s protocol (BD Biosciences). Total protein concentration in BALF was assessed using a bicinchoninic acid assay (Thermo Fisher) according to the manufacturer’s instructions. Thrombin-antithrombin complex (TATc) levels were determined by ELISA in citrated plasma and BALF according to the manufacturer’s instructions (Affinity Biologicals). D-dimer formation and cross-linked fibrin levels were determined by Western blot in lung homogenates using rabbit anti-mouse fibrinogen antibody (Sanbio) (17). Fibrinogen depletion in citrated plasma was confirmed by Western blot using the same antibody. Western blot results were quantified by densitometry analysis using Adobe Photoshop version 24.3.0 (Adobe).

### 2.8 RT-PCR analysis of bronchial epithelial brushes

Total RNA isolation from bronchial brushes, cDNA synthesis, and qPCR were performed as previously described (20). Data were analyzed with LinRegPCR software based on PCR efficiency values derived from amplification curves. *Hprt* mRNA levels were used for normalization. All primers are listed in **Supplementary Table S1**.

### 2.9 Histopathology and immunohistochemistry of the lung

Lungs were processed and scored by an independent pathologist blinded for experimental groups, as described (21). The following parameters were scored on a scale of 0 (absent), 1 (mild), 2 (moderate), and 3 (severe): interstitial inflammation, endotheliitis, bronchitis, edema, pleuritis, and confluence. The pathology score was expressed as the sum of the scores for each parameter. Bronchitis was further analyzed for the absence or presence thereof through neutrophil staining using a Ly6G monoclonal antibody (clone 1A8; Biolegend), as described (19).

### 2.10 Statistical analysis

Non-parametric variables were analyzed by Kruskall-Wallis test when comparing multiple groups, and by Mann-Whitney U when comparing two groups within an experiment. A chi-square test was performed for analysis of CFUs and bronchitis (absent or present). Analysis was performed using GraphPad Prism version 9 (Graphpad Software, San Diego, CA). Statistical significance is shown as *p<0.05, **p<0.01, ***p<0.001, ****p<0.0001.

## 3 Results

### 3.1 Topical flagellin treatment induces neutrophil influx in the lung during antibiotic-treated *K. pneumoniae* infection

Previous studies demonstrated that i.n. administration of the TLR5 agonist flagellin, either by itself or during antibiotic-treated pneumococcal pneumonia, exerts immunostimulatory effects in the lung (9, 10, 22). Here we investigated whether i.n. flagellin modulates pulmonary immune responses during antibiotic-treated pneumonia evoked by *K. pneumoniae.*

**Figure 1:**
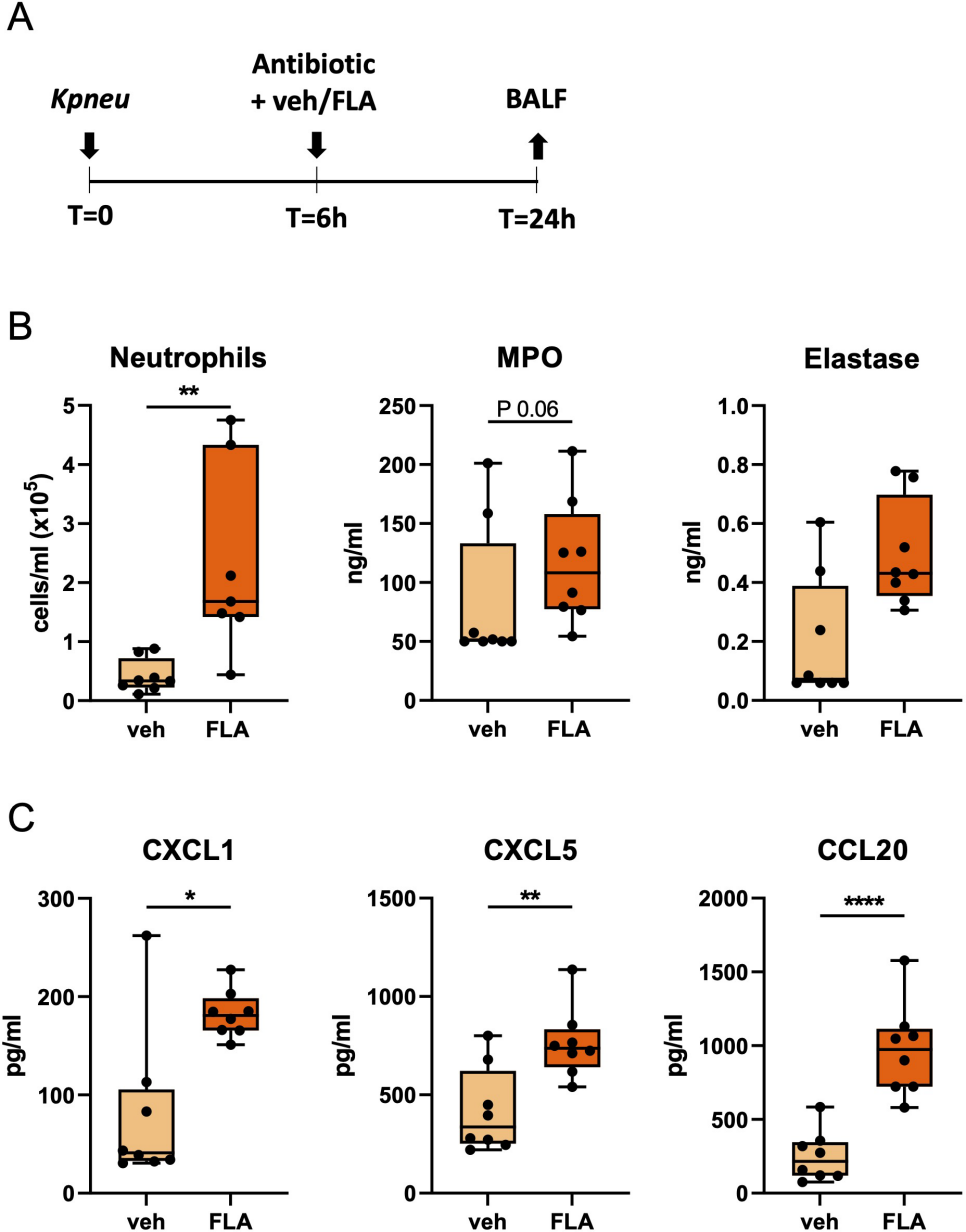
Topical flagellin treatment induces neutrophil influx in the lung during antibiotic-treated *Klebsiella* pneumonia. Mice were intranasally infected with antibiotic-susceptible *K. pneumoniae* (*Kpneu)*, treated with ceftriaxone and flagellin or vehicle (PBS) after 6 hours, and sacrificed after 24 hours **(A)**. Neutrophil counts, MPO, and elastase levels in bronchoalveolar lavage fluid (BALF) **(B)**. Chemokine levels in BALF **(C)**. Box and whiskers representing 8 mice per group. Differences were analyzed using Mann-Whitney test. *P<0.05, **P<0.01, ****P<0.0001. MPO, myeloperoxidase; veh, vehicle; FLA, flagellin.

In our initial experiments, mice were infected with antibiotic-susceptible *Kpneu* and treated 6 hours post-infection with ceftriaxone i.p. in combination with flagellin or vehicle i.n. An intranasal dose of 10 μg of flagellin was used in all our experiments, which was previously shown to trigger a robust innate immune response in the respiratory tract (10). After 24 hours, mice were sacrificed and BALF was harvested to assess neutrophil responses (**Figure 1A**). In comparison to vehicle treatment, flagellin induced more neutrophil influx in BALF, accompanied by a trend towards higher levels of the neutrophil degranulation products MPO and elastase (**Figure 1B**). In line with the increased neutrophil influx in the airways, mice treated with the combination of ceftriaxone and flagellin had higher levels of the neutrophil-attracting chemokines CXCL1, CXCL5 and CCL20 in BALF as compared to mice treated with ceftriaxone alone (**Figure 1C**). BALF concentrations of the pro-inflammatory cytokines TNF and IL-6, however, were not different between groups (**Supplementary Figure S3A**). Taken together, these data indicate that i.n. flagellin enhances neutrophil recruitment into the airways during antibiotic-treated *Kpneu* pneumonia, most likely at least in part through triggering the local release of neutrophil-attracting chemokines.

**Figure 2:**
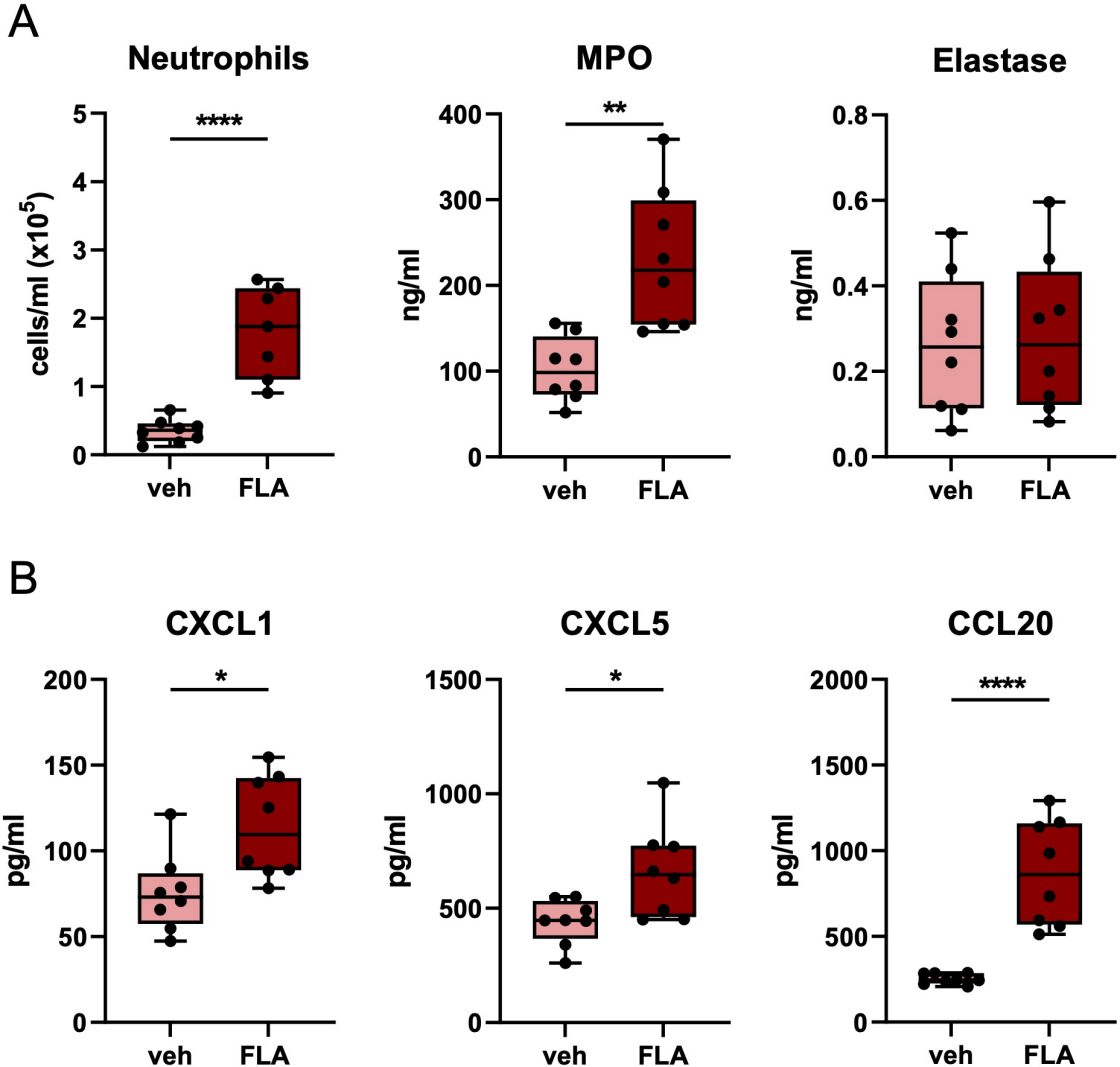
Topical flagellin treatment induces neutrophil influx in the lung during inadequately antibiotic-treated MDR-*Klebsiella* pneumonia. Mice were intranasally infected with multidrug-resistant-*K. pneumoniae* (MDR-*Kpneu*), treated with ceftriaxone and flagellin or vehicle after 6 hours, and sacrificed after 24 hours. Neutrophil counts, MPO, and elastase levels in BALF **(A)**. Chemokine levels in BALF **(B)**. Box and whiskers representing 8 mice per group. Differences were analyzed using Mann-Whitney test. *P<0.05, **P<0.01, ****P<0.0001. MPO, myeloperoxidase; veh, vehicle; FLA, flagellin.

Since antibiotic resistance of bacteria is an increasing problem, we next determined whether i.n. flagellin enhanced pulmonary immune responses in mice infected with antibiotic-resistant *Klebsiella*. Therefore, mice were infected with the ceftriaxone-resistant MDR-*Kpneu* (11) and treated 6 hours post-infection with ceftriaxone i.p., and flagellin or vehicle i.n. In this setting flagellin largely reproduced its effects in pneumonia induced by ceftriaxone-susceptible *Kpneu* reported above: flagellin increased neutrophil influx in BALF, as well as BALF levels of MPO, CXCL1, CXCL5, and CCL20 24 hours after infection (**Figure 2**); TNF and IL-6 concentrations in BALF were not different between groups (**Supplementary Figure S3B**).

**Figure 3:**
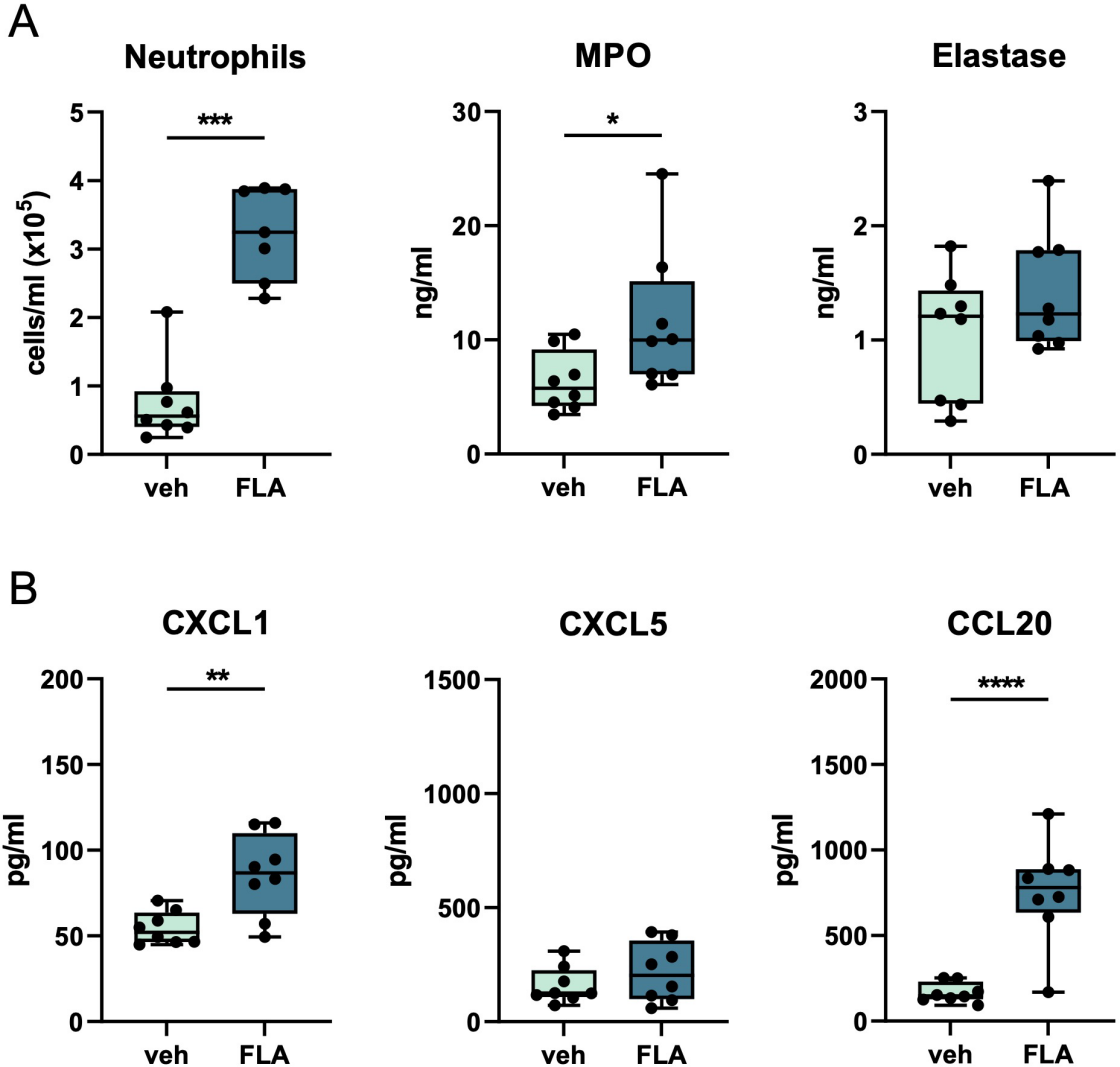
Topical flagellin treatment induces neutrophil influx in the lung during antibiotic-treated MDR-*Klebsiella* pneumonia. Mice were intranasally infected with MDR-*Kpneu*, treated with colistin and flagellin or vehicle after 6 hours, and sacrificed after 24 hours. Neutrophil counts, MPO, and elastase levels in BALF **(A)**. Chemokine levels in BALF **(B)**. Box and whiskers representing 8 mice per group. Differences were analyzed using Mann-Whitney test. *P<0.05, **P<0.01, ***P<0.001, ****P<0.0001. MPO, myeloperoxidase; veh, vehicle; FLA, flagellin.

Finally, we determined whether i.n. flagellin enhanced pulmonary immune responses in mice infected with antibiotic-resistant *Klebsiella* and treated with a ‘last resort’ antibiotic. Colistin, also known as Polymyxin E, was recently reintroduced in the clinic, despite its toxicity, particularly to treat MDR gram-negative pathogens such as *K. pneumoniae* (23). Mice were infected with MDR-*Kpneu* i.n. and 6 hours later treated with colistin i.p. in combination with flagellin or vehicle i.n. Similar to our earlier findings, neutrophil influx, MPO, CXCL1, and CCL20 levels in BALF were higher after i.n. flagellin treatment as compared to administration of vehicle (**Figure 3**). TNF and IL-6 levels in BALF (**Supplementary Figure S3C**) were also increased in this experiment.

In conclusion, these studies reveal that topical flagellin treatment enhances neutrophil influx during antibiotic-treated *K. pneumoniae*-evoked pneumonia in mice, irrespective of the antibiotic sensitivity of the bacteria.

### 3.2 Flagellin as adjunctive therapy to antibiotics reduces bacterial dissemination

**Figure 4:**
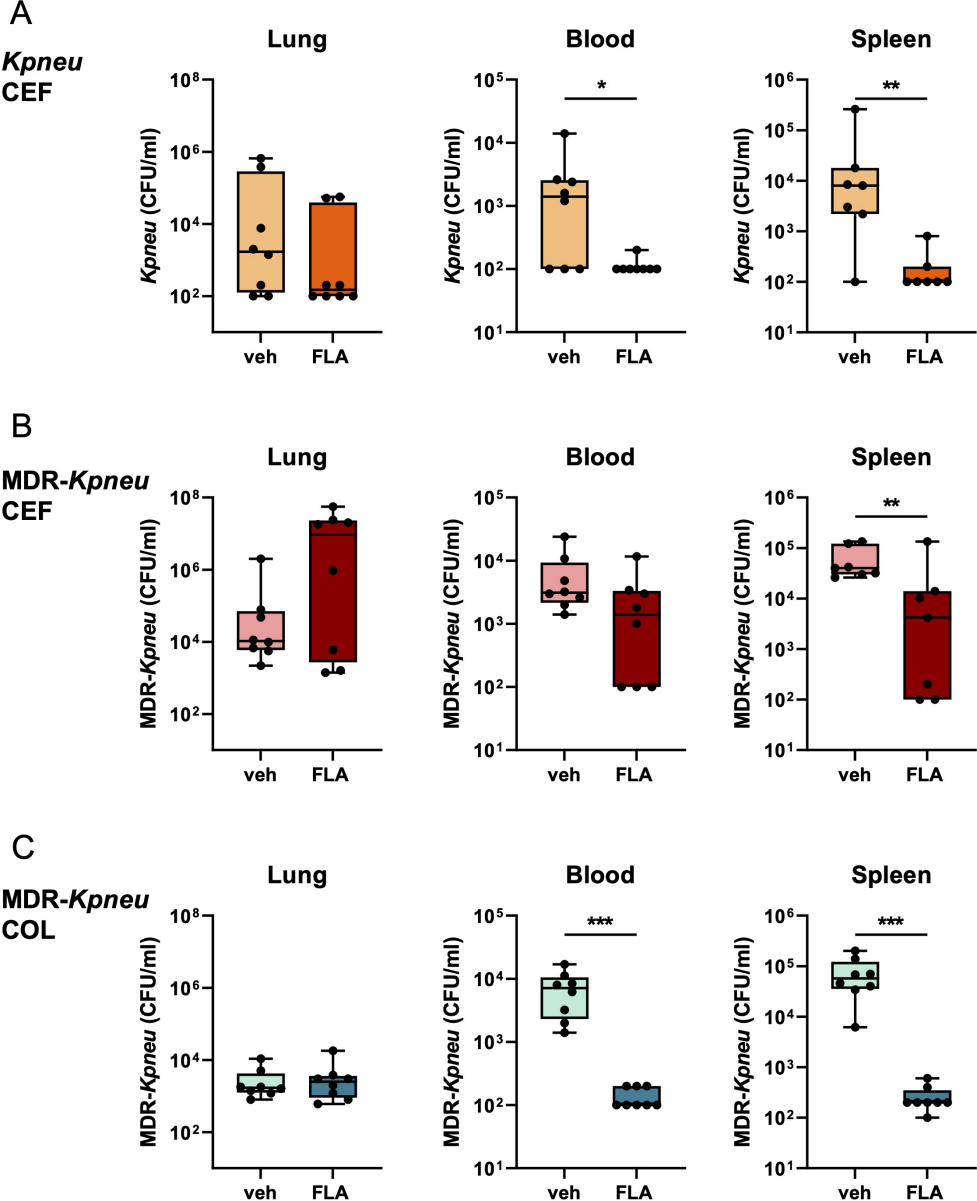
Flagellin as adjunctive therapy to antibiotics reduces bacterial dissemination. Mice were infected with antibiotic-susceptible *K. pneumoniae* (*Kpneu*) and treated with ceftriaxone and flagellin or vehicle after 6 hours **(A)**. Alternatively, mice were infected with MDR*-K. pneumoniae* (MDR-*Kpneu*) and treated with ceftriaxone and flagellin or vehicle **(B)**, or with colistin and flagellin or vehicle after 6 hours **(C)**. Endpoint of infection was after 24 hours. CFUs in lung, blood, and spleen were measured. Box and whiskers representing 8 mice per group. Differences were analyzed using Mann-Whitney test. *P<0.05, **P<0.01, ***P<0.001. CFU, Colony Forming Units; veh, vehicle; FLA, flagellin; CEF, ceftriaxone; COL, colistin.

We next determined whether topical flagellin treatment impacted the number of *Klebsiella* in lungs, blood, and spleen. The combination of ceftriaxone and flagellin treatment during *Kpneu* infection resulted in lower bacterial counts in blood and spleen (**Figure 4A**), compared to mice that were treated with ceftriaxone and vehicle. However, despite the enhanced influx of neutrophils in the airways of flagellin-treated mice, bacterial counts in the lungs did not differ between the groups. Likewise, bacterial counts of ceftriaxone-resistant MDR-*Kpneu* in spleens of mice treated with ceftriaxone and flagellin were lower than those after treatment with ceftriaxone alone; in blood, flagellin tended to lower bacterial numbers (**Figure 4B**). However, adjunctive treatment with flagellin did not impact CFUs in the lungs in this model. Similarly, after treatment with colistin, MDR-*Kpneu* numbers in blood and spleen were reduced by the addition of flagellin, whilst flagellin did not modify bacterial loads in the lungs (**Figure 4C**). Of note, plasma TNF and IL-6 levels paralleled bacterial loads in blood, except for TNF in *Kpneu* inoculated mice which were slightly above detection level in both groups (**Supplementary Figures S3D–F**). To determine whether the flagellin-mediated effect on bacterial dissemination persisted beyond the 24 hour time point, mice were infected with either *Kpneu* or MDR-*Kpneu*, treated with ceftriaxone and flagellin or vehicle 6 and 28 hours post-infection and bacterial loads were analyzed 44 hours after bacterial inoculation. In mice infected with antibiotic-susceptible *Kpneu*, ceftriaxone treatment eradicated nearly all bacteria from the systemic compartment, leaving no additional effect of flagellin to be determined (**Supplementary Figure S4A**). In contrast, in mice infected with MDR-*Kpneu*, bacterial loads in blood and spleen of vehicle and flagellin treated mice were similar (**Supplementary Figure S4B**). While these results may suggest that prolonged flagellin treatment is ineffective to limit bacterial dissemination, the similar CFU may also result from converged outgrowth of systemic bacteria that were already disseminated at an earlier stage.

Together, these results indicate that despite an augmented immune response in the lung, flagellin in combination with antibiotic treatment does not impact bacterial loads at this site during *K. pneumoniae-*induced pneumonia. However, flagellin consistently reduces bacterial dissemination to blood and spleen after 24 hours of infection with (MDR-)*Kpneu*; this effect was more pronounced when bacteria were susceptible to the administered antibiotic.

### 3.3 Adjunctive flagellin treatment does not increase the lung epithelial barrier during *K. pneumoniae*-evoked pneumonia

To study the mechanism underlying the effect of flagellin on bacterial dissemination, we first analyzed whether flagellin improved the lung epithelial barrier and thereby reduced dissemination. To assess whether flagellin improved intracellular connections between epithelial cells we analyzed the effect of flagellin treatment on mRNA expression of genes related to barrier function (24). mRNA levels of the tight junction genes *Tjp1*, *Ocln*, and *Cdh1* in bronchial epithelial brushes, however, did not differ between flagellin or vehicle-treated mice (**Supplementary Figure S5**).

**Figure 5:**
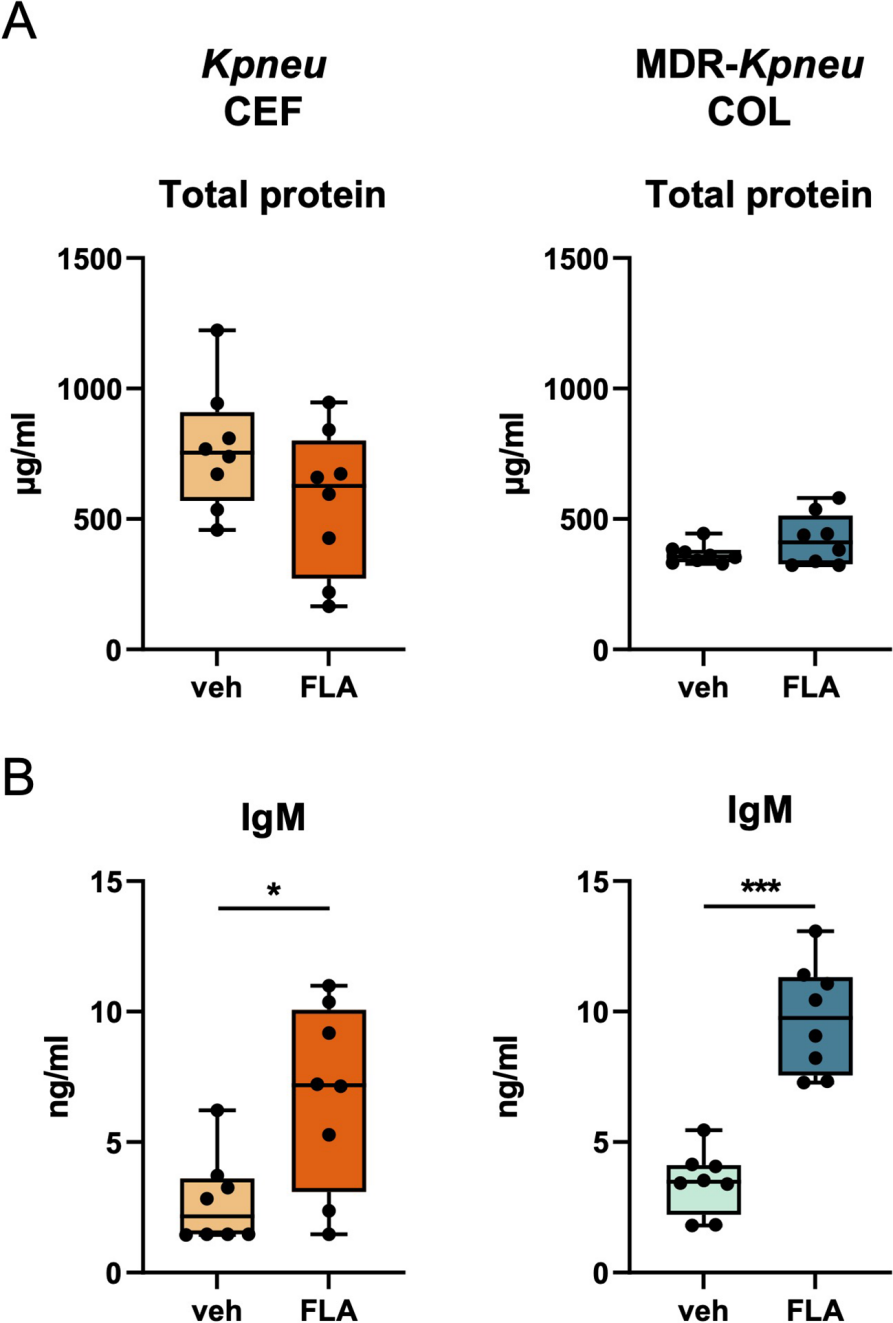
Adjunctive flagellin treatment does not increase the lung epithelial barrier during *Klebsiella* pneumonia. Mice were intranasally infected with antibiotic-susceptible *K. pneumoniae* (*Kpneu*) and treated with ceftriaxone and flagellin or vehicle after 6 hours. Alternatively, mice were infected with MDR*-K. pneumoniae* (MDR-*Kpneu*) and treated with colistin and flagellin or vehicle after 6 hours. Endpoint of infection was after 24 hours. Total protein concentration **(A)** and IgM concentration **(B)** in BALF. Box and whiskers representing 8 mice per group. Differences were analyzed using Mann-Whitney test. *P<0.05, ***P<0.001. veh, vehicle; FLA, flagellin; CEF, ceftriaxone; COL, colistin.

To further evaluate the lung epithelial barrier, we measured total protein and IgM concentrations in BALF. Flagellin did not affect alveolar total protein levels of *Kpneu-*infected mice treated with ceftriaxone or MDR-*Kpneu-*infected mice treated with colistin (**Figure 5A**). In contrast, IgM levels in BALF – a common marker of plasma leakage into the lungs and thus barrier function – were increased in flagellin treated mice (**Figure 5B**).

Taken together, these results suggest that adjunctive flagellin treatment does not ameliorate the lung epithelial barrier during *K. pneumoniae* infection, but rather modestly enhances protein leak.

### 3.4 Flagellin-induced fibrin formation in the lung has a modest effect on bacterial dissemination of MDR-*K. pneumoniae*

Next, we hypothesized that adjunctive flagellin reduced bacterial dissemination through activation of local coagulation during *K. pneumoniae* infection. Our group previously showed that blood coagulation is an important mechanism for the host defense and containment of *K. pneumoniae* (17, 25). To this end, we measured thrombin concentrations in BALF of (MDR-)*Kpneu*-infected mice, using a TATc ELISA. Adjunctive flagellin treatment increased BALF TATc levels in all groups that were treated with an antibiotic the *Klebsiella* strain was susceptible to: i.e. *Kpneu* with ceftriaxone and MDR-*Kpneu* with colistin (**Supplementary Figure S6**).

**Figure 6:**
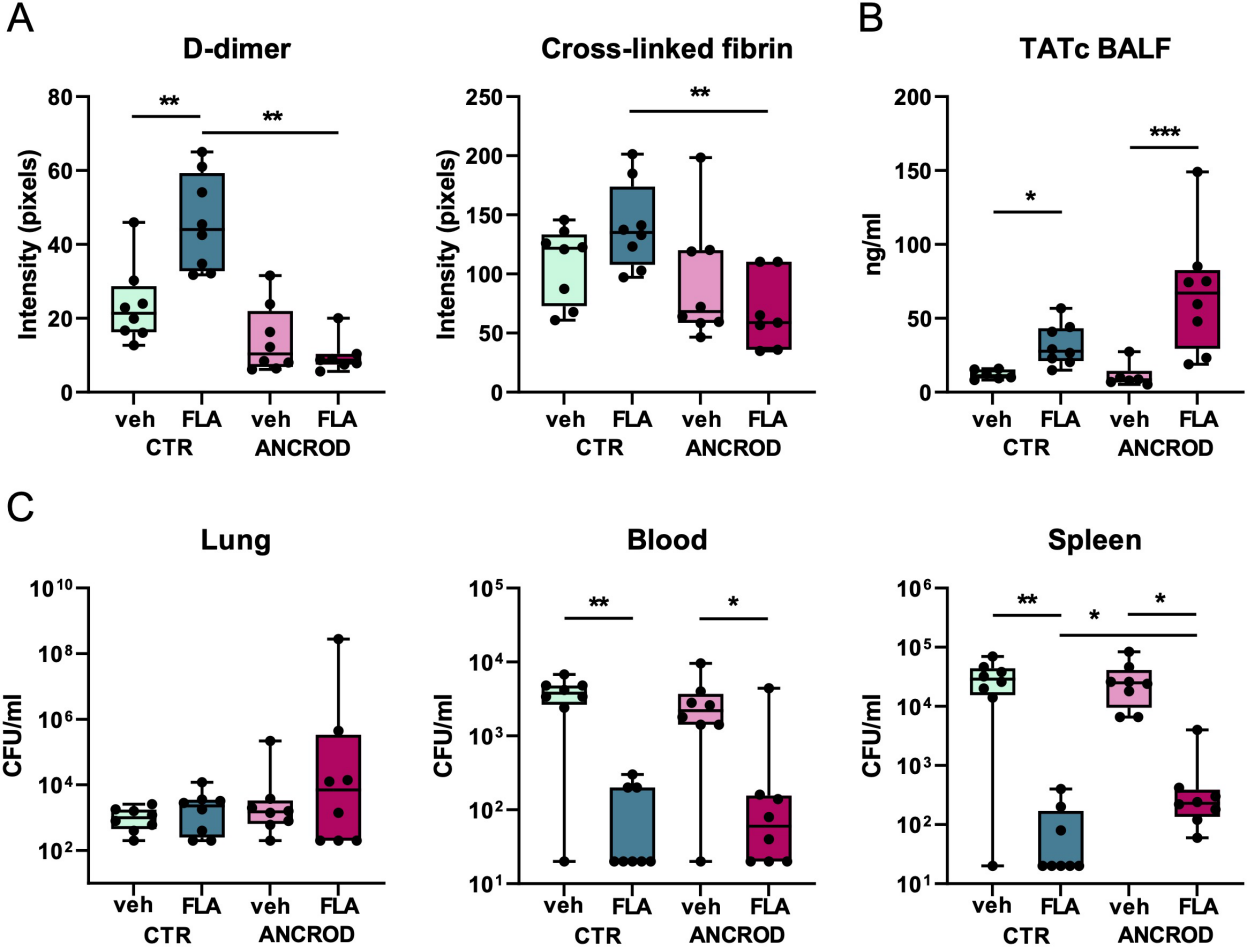
Flagellin-induced fibrin formation in the lung has a modest effect on bacterial dissemination of MDR-*K. pneumonia.* Mice were pre-treated with ancrod (to deplete fibrinogen) or saline, (control), 24 hours and immediately before intranasal infection with MDR*-K. pneumoniae.* Subsequent treatment with colistin and flagellin or vehicle was administered after 6 hours. Mice were sacrificed after 24 hours. Mean intensity of D-dimer and cross-linked fibrin from three blots combined **(A)**, TATc levels in BALF **(B)**; CFU counts in lung, blood, and spleen **(C)**. Box and whiskers representing 8 mice per group. Differences between groups were analyzed using Mann-Whitney test. For analysis of the presence or absence of CFUs in blood and spleen, a chi-square test was used. *P<0.05, **P<0.01, ***P<0.001. CFU, Colony Forming Units; veh, vehicle; FLA, flagellin; CTR, control.

To determine whether flagellin-induced coagulation activation is crucial for reducing *Klebsiella* dissemination, we investigated the effect of ancrod, a fibrinogen-depleting agent (17, 26) in our model. We hypothesized that if flagellin treatment inhibits *Klebsiella* spreading from the lungs through induction of local coagulation, this beneficial effect of flagellin would be prevented by ancrod-mediated fibrinogen depletion. Therefore, mice were depleted of fibrinogen by administration of ancrod (control mice were treated with saline) prior to i.n. inoculation with MDR-*Kpneu* and subsequent treatment with colistin and flagellin or vehicle. To confirm the effect of ancrod on local activation of the coagulation system, we performed Western blot analysis for the fibrin degradation product D-dimer and for cross-linked fibrin in lung homogenates. Flagellin induced an increase in D-dimer and cross-linked fibrin levels in the lung, which was prevented in ancrod-treated mice (**Supplementary Figure S7**, **Figure 6A**). Flagellin-induced rise in BALF TATc levels was more pronounced after ancrod treatment, as thrombin accumulates in the lung without its substrate fibrinogen to be converted to fibrin (**Figure 6B**).

Reduced fibrin formation in ancrod-treated mice had limited impact on the effect of flagellin on bacterial dissemination (**Figure 6C**). While flagellin again strongly reduced bacterial loads in blood and spleen, ancrod only modestly reversed this flagellin effect in the spleen. In lungs, neither flagellin nor ancrod impacted bacterial counts.

These data suggest that flagellin-induced local coagulation activation contributes only modestly to the flagellin-mediated decrease in bacterial dissemination during *K. pneumoniae-*evoked pneumonia.

### 3.5 Flagellin-induced reduction of bacterial dissemination during MDR-*K.pneumoniae* infection is dependent on neutrophils

**Figure 7:**
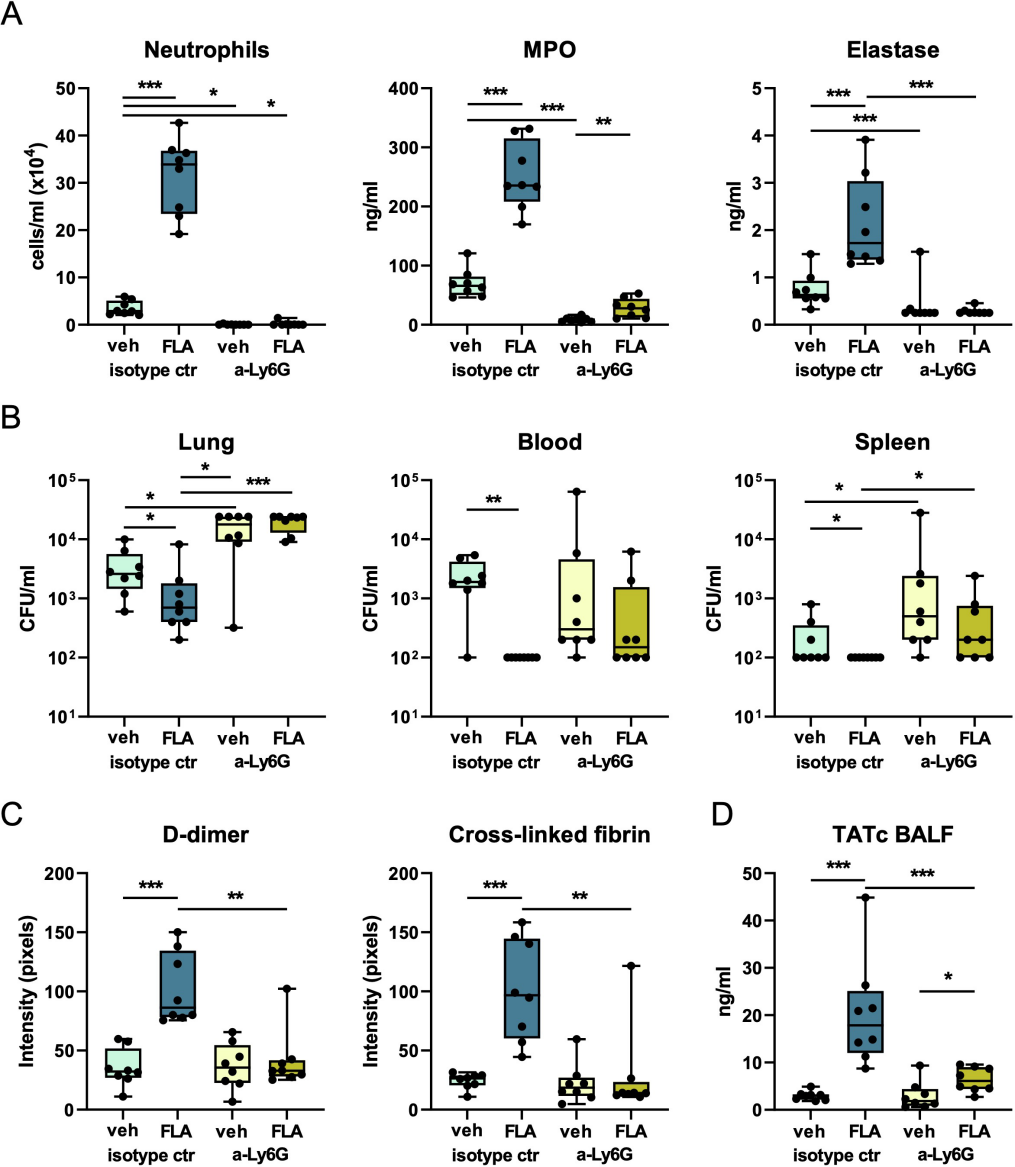
Flagellin-induced reduction of bacterial dissemination during MDR-*K. pneumoniae* infection is dependent on neutrophils. Mice were pre-treated with anti-Ly6G (to deplete neutrophils)or isotype control 48, 24, and 3 hours before infection with MDR-*Kpneu*. In addition, all mice received anti-rat κ immunoglobulin light chain antibody as a secondary antibody 46 and 1 hour prior to bacterial inoculation. Subsequent treatment with colistin and flagellin or vehicle was administered after 6 hours. Mice were sacrificed after 24 hours. Neutrophil numbers, MPO, and elastase levels in BALF **(A)**. CFU counts in lung, blood, and spleen **(B)**. Mean intensity of D-dimer and cross-linked fibrin from three blots combined **(C)**, and TATc levels in BALF **(D)**. Box and whiskers representing 8 mice per group. Differences between groups were analyzed using Mann-Whitney test. *P<0.05, **P<0.01, ***P<0.001. Of note, lung CFU in anti-Ly6G treated mice reached the upper limit of detection (2.4x10^4^ CFU/ml) in 4/8 vehicle- and 4/8-flagellin-treated mice. MPO, myeloperoxidase; CFU, Colony Forming Units; veh, vehicle; FLA, flagellin.

To study the contribution of flagellin-induced neutrophil recruitment to the lung in bacterial dissemination, mice were depleted of neutrophils before infection by administration of anti-Ly6G antibody, whereas control mice were treated with an isotype-matched control antibody (18). Hereafter, mice were infected i.n. with MDR-*Kpneu* and 6 hours later treated with colistin, and flagellin or vehicle. Flow cytometry analysis showed that neutrophils were indeed depleted from BALF 24 hours after infection (**Figure 7A**). In agreement, MPO and elastase levels in BALF were significantly decreased after neutrophil depletion (**Figure 7A**). Moreover, although overall lung pathology scores were not different between groups, analysis of bronchitis in the lungs of isotype-treated (neutrophil-sufficient) mice showed (intra-)bronchial accumulation of neutrophils after flagellin treatment as compared to vehicle-treated mice (**Supplementary Figure S8**), which was not observed in neutrophil depleted mice, as expected. Bacterial counts in the lungs of neutrophil-depleted mice were higher as compared to isotype-treated mice (**Figure 7B**). Flagellin decreased CFU counts in blood and spleen of mice pre-treated with isotype control antibody (**Figure 7B**), confirming the results shown in **Figures 4C** and **6C**. Strikingly, flagellin did not diminish bacterial loads in the blood and spleen of neutrophil-depleted mice (**Figure 7B**), and the number of culture-positive samples was higher in flagellin-treated neutrophil-depleted mice as compared to flagellin-treated neutrophil-sufficient mice for both blood and spleen (P<0.05 in both cases using Chi-square), indicating that neutrophils limit bacterial dissemination in this setting.

Since neutrophils are known to contribute to coagulation activation and immunothrombosis (27), we assessed whether neutrophil depletion impacted the flagellin-induced local coagulation responses during antibiotic-treated MDR-*Kpneu* pneumonia. Analysis of D-dimer and cross-linked fibrin in lung homogenates demonstrated that neutrophil depletion abrogated the flagellin-induced increase in these coagulation markers (**Figure 7C**, **Supplementary Figure S9**). In line with these results, neutrophil depletion also reduced flagellin-induced TATc levels in BALF (**Figure 7D**).

Taken together, these data indicate that neutrophils play an important role in limiting bacterial dissemination and that flagellin ameliorates this process by triggering the recruitment, activation, and bronchial accumulation of neutrophils to the primary site of infection.

## 4 Discussion

This study aimed to investigate the immune-enhancing effect of flagellin in the search for new adjunctive treatment modalities that aid antibiotics in the management of respiratory tract infection by MDR-*K. pneumoniae*. To this end, we treated mice with established pneumonia, evoked by either antibiotic-susceptible or MDR-*Kpneu*, concurrently with systemic antibiotics and topical flagellin (or vehicle as control). The results of our study reveal that flagellin treatment induced the release of neutrophil chemoattractants and increased neutrophil influx, as well as neutrophil activation, in the lung after *Kpneu* or MDR-*Kpneu* infection and treatment with different types of antibiotics, but did not impact antibacterial defense at this site. Strikingly, we found that topical adjunctive flagellin treatment reduced bacterial dissemination to systemic compartments, most effectively when bacteria were susceptible to the given antibiotic, and established that this effect depended on enhanced local neutrophil responses.

It is well established that neutrophil recruitment to the lung is crucial for host defense against *K. pneumoniae* at this site. Studies with mice depleted of neutrophils demonstrated enhanced outgrowth of *Klebsiella* in the lung during experimentally induced pneumonia (28, 29). Likewise, MPO-deficient mice showed a greater bacterial burden in the lung, indicating that neutrophil degranulation products are instrumental in controlling local *K. pneumoniae* infection (30). In line with these reports, studies in mice with boosted neutrophil responses, evoked either by chemokine overexpression (31) or immunostimulatory agents (29, 32) demonstrated reduced *K. pneumoniae* numbers in the lung. In the current study, we observed a marked influx of neutrophils and higher levels of neutrophil degranulation products in BALF after flagellin treatment. Surprisingly, we consistently did not detect a decrease in bacterial counts at the primary site of infection. The discrepancy between the results of our study and the latter reports may be explained by differences in the timing of neutrophil activation (prior to bacterial inoculation versus after onset of pneumonia in the current study) (29–32).

An unexpected finding in our neutrophil depletion experiment was that ‘control’ mice treated with isotype control antibodies displayed lower bacterial numbers in the spleen as compared to mice in previous experiments, but not in other organs. Although we do not have direct proof, we speculate that these differences may result from the additional antibody treatment prior to the bacterial inoculation. Although to our knowledge there have been no reports indicating that these ‘control’ antibodies modulate the immune response in mice, we cannot exclude an effect hereof on bacterial clearance in the spleen. Further studies are required to examine the effect of this treatment in the *Klebsiella* pneumosepsis model. Another unexpected finding was the lack of increased dissemination of *Klebsiella* in blood and spleen of mice depleted of neutrophils as compared to neutrophil-sufficient mice, given that this treatment is associated with enhanced outgrowth of bacteria in systemic organs (28, 29). An important difference with these previous studies, however, is that in the current study all mice were also treated systemically with antibiotics to which the bacteria were susceptible. While the antibiotic treatment may not be optimal to reduce CFU in the lung of neutrophil depleted mice to the level of neutrophil-sufficient control mice, it may be more effective in systemic organs.

Of interest, we found neutrophil accumulation in and around the bronchi in flagellin-treated mice, which possibly can partially explain the effect of flagellin on bacterial dissemination. Notably, increases in neutrophil influx in the lung following flagellin treatment could also be harmful, as excessive neutrophil recruitment may cause immunopathology and thereby increase disease severity (33, 34). In the current study, however, we did not observe augmented lung damage in flagellin-treated mice as revealed by analysis of lung pathology (**Supplementary Figure S8**). These latter findings are in agreement with an earlier study with combined antibiotic and flagellin treatment in *S. pneumoniae* infection models (13).

Previously, we demonstrated that fibrin formation in the lungs contributes to protective immunity during *K. pneumoniae-*evoked pneumonia (17). Furthermore, *in vitro* experiments showed that inhibition of thrombin-induced fibrin polymerization reduced *K. pneumoniae* outgrowth in whole blood and that this effect was largely dependent on neutrophils (17). This protective role of coagulation in combination with platelets and innate immune cells, including neutrophils, in host defense against *K. pneumoniae* and a variety of other pathogens has been referred to as immunothrombosis (35, 36). In the current study, we found that prevention of flagellin-induced fibrin formation in the lung by ancrod only modestly impacted MDR-*Kpneu* numbers in the spleen, suggesting that local coagulation activation contributes to the flagellin-mediated decrease in bacterial dissemination. Considering that neutrophils played a major role in flagellin-induced local fibrin formation, these findings may indicate that neutrophils partially mediate their effect on bacterial spreading in flagellin-treated mice by an effect on local fibrin formation.

Our study corroborates various studies showing that flagellin is a potent inducer of neutrophil-attracting chemokines in the lungs (9, 13, 22). It is well known that flagellin induces chemokine secretion by airway epithelial cells by activation of TLR5, which is most abundantly expressed on the surface of these cells (9, 37). Previously, we demonstrated that mRNA expression of *Cxcl1*, *Cxcl5*, and *Ccl20* was upregulated in bronchial epithelial cells 2 hours after intranasal flagellin administration to mice, followed by a gradual and robust increase in neutrophil recruitment into the lung (22). Of note, however, murine dendritic cells express TLR5 as well and can be stimulated by flagellin via the TLR5 signaling pathway (38). Dendritic cells are also abundant in the lung and can protrude the epithelial cell layer to detect pathogens and aid in host defense (39).

Pulmonary epithelial cells play an important role in the barrier function of the lung (7, 40, 41). Previously, it was shown that TLR2 stimulation of bronchial epithelial cells increased claudin-1 and ZO-1 expression and tight junctional barrier function (42). Our study, however, did not demonstrate reduced protein leakage in the lung lumen nor increased expression of tight junction proteins, suggesting that the epithelial barrier function was not improved by flagellin treatment.

Whether our findings are applicable to the treatment of pneumonia evoked by other (antibiotic-resistant) bacteria requires further investigation. Previous studies showed that prophylactic or simultaneous treatment with topical flagellin was beneficial for host defense during lethal *Pseudomonas aeruginosa* and pneumococcal pneumonia, respectively (43, 44). Interestingly, the group of Sirard recently showed that in mice with established pneumonia, evoked by *S. pneumoniae*, combined treatment with systemic antibiotics and intranasal flagellin improved survival and lowered bacterial outgrowth in the lungs and spleens of mice (10, 13). Furthermore, this group recently showed that development of antibiotic resistance during *S. pneumoniae* superinfection was prevented by the addition of flagellin (45), demonstrating flagellin’s potential for clinical use. Similar to the current study, these studies demonstrated increased levels of neutrophil-attracting chemokines along with augmented neutrophil influx in the lung. Importantly, our study highlights the importance of neutrophil-mediated immunity induced by flagellin and reveals that flagellin is ineffective against *K. pneumoniae* in the absence of neutrophils. Since patients with hospital-acquired pneumonia are often immunocompromised or present with neutropenia (7) e.g. due to chemotherapy for treatment of malignancy, future studies should encompass immunocompromised settings relevant to human respiratory infections. While our study focused on the immunostimulatory effect of flagellin in the lung, there also have been studies to determine the effects in other organs such as the intestinal tract (9). Interestingly, systemically administered flagellin reduced susceptibility to intestinal colonization with vancomycin-resistant enterococcus in antibiotic-treated mice via increased expression of the antimicrobial peptide RegIIIγ in the intestinal epithelium amongst others (46). Whether systemically administered flagellin has potential in *Klebsiella* pneumosepsis remains to be determined. In view of the impact of antibiotic resistance on the efficacy of flagellin treatment, observed in the current study, rapid diagnostic testing of antimicrobial susceptibility may be considered in future clinical use of adjunctive flagellin therapy.

Besides flagellin, other TLR ligands may also be suitable for the induction of an immunostimulatory response in the lung that improves host defense against *K. pneumoniae*. We previously found that human airway epithelial cells, beside TLR5, also express TLR2 and TLR3 (47). Previously it was demonstrated that combined topical treatment with TLR2/6 and TLR9 ligands (and to a lesser extent also TLR3 and TLR7 ligands) improved host defense and protected mice against lethal bacterial lung infection (48, 49). The experimental design of these latter studies, however, differed markedly from our studies since the TLR ligands were administered 24 hours prior to bacterial inoculation and no antibiotics were applied. Future studies with models mimicking the clinical situation (delayed treatment during established infection) are required to assess whether other TLR ligands activating lung epithelial cells are useful for adjunctive therapy of pneumonia (40).

A limitation of our study is that our experiments with prolonged flagellin treatment in combination with antibiotics beyond 24 hours were inconclusive. Further experiments with more extensive flagellin treatment and a wider analysis of times are warranted to evaluate the efficacy of this adjunctive therapy on host defense and clinical outcome in the long-term. In this regard, it should be noted that survival studies are not allowed in this model according to Dutch legislation. Moreover, future studies should also encompass experiments with other, less virulent *Klebsiella* strains in immunocompromised mice to mimic a clinical relevant setting of hospital-acquired pneumonia (50). Furthermore, follow up studies without antibiotics, and with extensive titrations of antibiotics against MDR infections, are needed to elucidate the mechanism(s) by which flagellin augments host defense. Only after this expanded analysis it is possible to make a case for flagellin’s potential in topical treatment of *Klebsiella* unequivocally.

In summary, we here show that flagellin administration via the airways strongly reduces bacterial dissemination during pneumonia caused by antibiotic-susceptible or MDR-*K. pneumoniae*. The immunomodulatory effect of topical adjunctive flagellin treatment during ongoing respiratory infection is characterized by augmented chemokine release and recruitment of neutrophils in the lung and accompanied by activation of neutrophils and blood coagulation. Our results establish that the flagellin-induced neutrophil response in the lung is essential for limiting bacterial dissemination. These results indicate that topical adjunctive immunomodulatory drugs, such as flagellin, may ameliorate the treatment of ongoing pulmonary bacterial infections.

## Data Availability

The raw data supporting the conclusions of this article will be made available by the authors, without undue reservation.

## References

[1] Murray CJ Ikuta KS Sharara F Swetschinski L Robles Aguilar G Gray A. Global burden of bacterial antimicrobial resistance in 2019: a systematic analysis. Lancet. (2022) 399:629–55. doi: 10.1016/S0140-6736(21)02724-0 PMC8841637 35065702

[2] Mehrad B Clark NM Zhanel GG Lynch JP. Antimicrobial resistance in hospital-acquired gram-negative bacterial infections. Chest. (2015) 147:1413–21. doi: 10.1378/chest.14-2171 PMC4420185 25940252

[3] Song J Huh K Chung DR. Community-acquired pneumonia in the Asia-Pacific Region. Semin Respir Crit Care Med. (2016) 37:839–54. doi: 10.1055/s-0036-1592075 PMC7171710 27960208

[4] Ikuta KS Swetschinski LR Robles Aguilar G Sharara F Mestrovic T Gray AP. Global mortality associated with 33 bacterial pathogens in 2019: a systematic analysis for the Global Burden of Disease Study 2019. Lancet. (2022) 400:2221–48. doi: 10.1016/S0140-6736(22)02185-7 PMC9763654 36423648

[5] Angus DC van der Poll T. Severe sepsis and septic shock. N Engl J Med. (2013) 369:840–51. doi: 10.1056/NEJMra1208623 23984731

[6] Wyres KL Lam MMC Holt KE. Population genomics of Klebsiella pneumoniae. Nat Rev Microbiol. (2020) 18:344–59. doi: 10.1038/s41579-019-0315-1 32055025

[7] Torres A Cilloniz C Niederman MS Menéndez R Chalmers JD Wunderink RG. Pneumonia. Nat Rev Dis Primers. (2021) 7:25. doi: 10.1038/s41572-021-00259-0 33833230

[8] Torres LK Pickkers P van der Poll T. Sepsis-induced immunosuppression. Annu Rev Physiol. (2021) 84:157–81. doi: 10.1146/annurev-physiol-061121 34705481

[9] Vijayan A Rumbo M Carnoy C Sirard JC. Compartmentalized antimicrobial defenses in response to flagellin. Trends Microbiol. (2018) 26:423–35. doi: 10.1016/j.tim.2017.10.008 29173868

[10] Matarazzo L Casilag F Porte R Wallet F Cayet D Faveeuw C. Therapeutic synergy between antibiotics and pulmonary Toll-like receptor 5 stimulation in antibiotic-sensitive or -resistant pneumonia. Front Immunol. (2019) 10:723. doi: 10.3389/fimmu.2019.00723 31024555 PMC6465676

[11] Van der Weide H Ten Kate MT Vermeulen-De Jongh DMC van der Meijden A Wijma RA Boers SA. Successful high-dosage monotherapy of tigecycline in a multidrug-resistant Klebsiella pneumoniae pneumonia-septicemia model in rats. Antibiotics. (2020) 9:1–17. doi: 10.3390/antibiotics9030109 PMC7148456 32138210

[12] Qin W Liu Z van der Poll T De Vos AF. Induction of acute or disseminating bacterial pneumonia in mice and sampling of infected organs for studying the host response to bacterial pneumonia. Bio Protoc. (2022) 12:e4287. doi: 10.21769/BioProtoc.4287 PMC8769758 35118178

[13] Porte R Fougeron D Muñoz-Wolf N Tabareau J Georgel AF Wallet F. A toll-like receptor 5 agonist improves the efficacy of antibiotics in treatment of primary and influenza virus-associated pneumococcal mouse infections. Antimicrob Agents Chemother. (2015) 59:6064–72. doi: 10.1128/AAC.01210-15 PMC4576040 26195519

[14] Calame W Douwes-Idema AE van den Barselaar MT van Furth R Mattie H. Influence of cytostatic agents on the pulmonary defence of mice infected with Klebsiella pneumoniae and on the efficacy of treatment with ceftriaxone. J Infection. (1994) 29:53–66. doi: 10.1016/S0163-4453(94)95087-3 7963636

[15] Fergadaki S Renieris G Machairas N Sabracos L Droggiti DI Misiakos E. Efficacy of tigecycline alone or in combination for experimental infections by KPC carbapenemase-producing Klebsiella pneumoniae. Int J Antimicrob Agents. (2021) 58:106384. doi: 10.1016/j.ijantimicag.2021.106384 34161789

[16] Biedma ME Cayet D Tabareau J Rossi AH Ivičak-Kocjan K Moreno G. Recombinant flagellins with deletions in domains D1, D2, and D3: Characterization as novel immunoadjuvants. Vaccine. (2019) 37:652–63. doi: 10.1016/j.vaccine.2018.12.009 30583910

[17] Claushuis TAM de Stoppelaar SF Stroo I Roelofs JJTH Ottenhoff R van der Poll T. Thrombin contributes to protective immunity in pneumonia-derived sepsis via fibrin polymerization and platelet-neutrophil interactions. J Thromb Haemost. (2017) 15:744–57. doi: 10.1111/jth.13625 28092405

[18] Boivin G Faget J Ancey P-B Gkasti A Mussard J Engblom C. Durable and controlled depletion of neutrophils in mice. Nat Commun. (2020) 11:2762. doi: 10.1038/s41467-020-16596-9 32488020 PMC7265525

[19] Liu Z De Porto APNA De Beer R Roelofs JJTH De Boer OJ Florquin S. Bruton’s Tyrosine Kinase in Neutrophils Is Crucial for Host Defense against Klebsiella pneumoniae. J Innate Immun. (2022) 15:1–15. doi: 10.1159/000524583 35537415 PMC10643901

[20] Ferreira BL Ramirez-Moral I Otto NA Salomão R de Vos AF van der Poll T. The PPAR-γ agonist pioglitazone exerts proinflammatory effects in bronchial epithelial cells during acute Pseudomonas aeruginosa pneumonia. Clin Exp Immunol. (2022) 207:370–7. doi: 10.1093/cei/uxab036 PMC9113127 35553637

[21] Meijer MT de Vos AF Scicluna BP Roelofs JJ Abou Fayçal C Orend G. Tenascin-C deficiency is associated with reduced bacterial outgrowth during Klebsiella pneumoniae-evoked pneumosepsis in mice. Front Immunol. (2021) 12:600979. doi: 10.3389/fimmu.2021.600979 33776992 PMC7990887

[22] Qin W Brands X van ‘t Veer C de Vos AF Scicluna BP van der Poll T. Flagellin induces innate immune genes in bronchial epithelial cells *in vivo*: Role of TET2. Scand J Immunol. (2021) 94:4–7. doi: 10.1111/sji.13046 33904193

[23] Ahmed MAEE Doi Y Tian G Zhong L Shen C Yang Y. Colistin and its role in the Era of antibiotic resistance: an extended review. Emerg Microbes Infect. (2020) 9:868–85. doi: 10.1080/22221751.2020.1754133 PMC7241451 32284036

[24] Qin W Brands X van’t Veer C de Vos AF Scicluna BP van der Poll T. Bronchial epithelial tet2 maintains epithelial integrity during acute pseudomonas aeruginosa pneumonia. Infect Immun. (2021) 89:1–10. doi: 10.1128/IAI.00603-20 PMC7927922 33046509

[25] Perlee D de Beer R Florquin S van der Poll T van ‘t Veer C de Vos AF. Caspase-11 contributes to pulmonary host defense against Klebsiella pneumoniae and local activation of coagulation. Am J Physiol Lung Cell Mol Physiol. (2020) 319:L105–14. doi: 10.1152/ajplung.00422.2019 32401674

[26] Mckillop C Edgar W Forbes CD Prentice CRM. Possible pathway for formation of fibrin degradation products during ancrod therapy. Nature. (1975) 255:638–40. doi: 10.1038/255638a0 1134556

[27] Iba T Levi M Levy JH. Intracellular communication and immunothrombosis in sepsis. J Thromb Haemostasis. (2022) 20:2475–84. doi: 10.1111/jth.15852 PMC9804233 35979601

[28] Xiong H Carter RA Leiner IM Tang YW Chen L Kreiswirth BN. Distinct contributions of neutrophils and CCR2+ monocytes to pulmonary clearance of different Klebsiella pneumoniae strains. Infect Immun. (2015) 83:3418–27. doi: 10.1128/IAI.00678-15 PMC4534658 26056382

[29] Ramirez-Moral I Blok DC Bernink JH Garcia-Laorden MI Florquin S Boon L. Interleukin-33 improves local immunity during Gram-negative pneumonia by a combined effect on neutrophils and inflammatory monocytes. J Pathol. (2021) 253:374–83. doi: 10.1002/path.5601 PMC7986604 33305354

[30] Hirche TO Gaut JP Heinecke JW Belaaouaj A. Myeloperoxidase plays critical roles in killing Klebsiella pneumoniae and inactivating neutrophil elastase: effects on host defense1. J Immunol. (2005) 174:1557–65. doi: 10.4049/jimmunol.174.3.1557 15661916

[31] Tsai WC Strieter RM Wilkowski JM Bucknell KA Burdick MD Lira SA. Lung-specific transgenic expression of KC enhances resistance to Klebsiella pneumoniae in mice. J Immunol. (1998) 161:2435–40. doi: 10.4049/jimmunol.161.5.2435 9725241

[32] Deng JC Moore TA Newstead MW Zeng X Krieg AM Standiford TJ. CpG oligodeoxynucleotides stimulate protective innate immunity against pulmonary Klebsiella infection1. J Immunol. (2004) 173:5148–55. doi: 10.4049/jimmunol.173.8.5148 PMC3001228 15470059

[33] Palmer CS Kimmey JM. Neutrophil recruitment in pneumococcal pneumonia. Front Cell Infect Microbiol. (2022) 12:894644. doi: 10.3389/fcimb.2022.894644 35646729 PMC9136017

[34] Iwasaki A Foxman EF Molony RD. Early local immune defences in the respiratory tract. Nat Rev Immunol. (2017) 17:7–20. doi: 10.1038/nri.2016.117 27890913 PMC5480291

[35] de Stoppelaar SF van ‘t Veer C Claushuis TAM Albersen BJA Roelofs JJTH van der Poll T. Thrombocytopenia impairs host defense in gram-negative pneumonia-derived sepsis in mice. Blood. (2014) 124:3781–90. doi: 10.1182/blood-2014-05-573915 PMC4263985 25301709

[36] Engelmann B Massberg S. Thrombosis as an intravascular effector of innate immunity. Nat Rev Immunol. (2013) 13:34–45. doi: 10.1038/nri3345 23222502

[37] Van Maele L Fougeron D Janot L Didierlaurent A Cayet D Tabareau J. Airway structural cells regulate TLR5-mediated mucosal adjuvant activity. Mucosal Immunol. (2014) 7:489–500. doi: 10.1038/mi.2013.66 24064672

[38] Didierlaurent A Ferrero I Otten LA Dubois B Reinhardt M Carlsen H. Flagellin promotes myeloid differentiation factor 88-dependent development of th2-type response. J Immunol. (2004) 172:6922–30. doi: 10.4049/jimmunol.172.11.6922 15153511

[39] Lambrecht BN Prins JB Hoogsteden HC. Lung dendritic cells and host immunity to infection. Eur Respir J. (2001) 18:692–704. doi: 10.1183/09031936.01.18040692 11716176

[40] Leiva-Juárez MM Kolls JK Evans SE. Lung epithelial cells: therapeutically inducible effectors of antimicrobial defense. Mucosal Immunol. (2018) 11:21–34. doi: 10.1038/mi.2017.71 28812547 PMC5738267

[41] Invernizzi R Lloyd CM Molyneaux PL. Respiratory microbiome and epithelial interactions shape immunity in the lungs. Immunology. (2020) 160:171–82. doi: 10.1111/imm.13195 PMC7218407 32196653

[42] Ragupathy S Esmaeili F Paschoud S Sublet E Citi S Borchard G. Toll-like receptor 2 regulates the barrier function of human bronchial epithelial monolayers through atypical protein kinase C zeta, and an increase in expression of claudin-1. Tissue Barriers. (2014) 2:e29166. doi: 10.4161/tisb.29166 25101232 PMC4117686

[43] Yu F Cornicelli MD Kovach MA Newstead MW Zeng X Kumar A. Flagellin stimulates protective lung mucosal immunity: role of cathelicidin-related antimicrobial peptide. J Immunol. (2010) 185:1142–9. doi: 10.4049/jimmunol.1000509 PMC3038689 20566829

[44] Muñoz N Van Maele L Marqués JM Rial A Sirard JC Chabalgoity JA. Mucosal administration of flagellin protects mice from Streptococcus pneumoniae lung infection. Infect Immun. (2010) 78:4226–33. doi: 10.1128/IAI.00224-10 PMC2950348 20643849

[45] Costa C Sirard J-C Gibson PS Veening J-W Gjini E Baldry M. Triggering toll-like receptor 5 signaling during pneumococcal superinfection prevents the selection of antibiotic resistance. J Infect Dis. (2024). doi: 10.1093/infdis/jiae239 PMC11566229 38716762

[46] Kinnebrew MA Ubeda C Zenewicz LA Smith N Flavell RA Pamer EG. Bacterial flagellin stimulates toll-like receptor 5-dependent defense against vancomycin-resistant Enterococcus infection. J Infect Dis. (2010) 201:534–43. doi: 10.1086/650203 PMC2811237 20064069

[47] van Linge CCA Hulme KD Peters-Sengers H Sirard JC Goessens WHF de Jong MD. Immunostimulatory effect of flagellin on MDR-Klebsiella-infected human airway epithelial cells. Int J Mol Sci. (2024) 25:309. doi: 10.3390/ijms25010309 PMC10778885 38203480

[48] Duggan JM You D Cleaver JO Larson DT Garza RJ Guzmán Pruneda FA. Synergistic interactions of TLR2/6 and TLR9 induce a high level of resistance to lung infection in mice. J Immunol. (2011) 186:5916–26. doi: 10.4049/jimmunol.1002122 PMC3654378 21482737

[49] Ware HH Kulkarni VV Wang Y Pantaleón García J Juarez ML Kirkpatrick CT. Inducible lung epithelial resistance requires multisource reactive oxygen species generation to protect against bacterial infections. PloS One. (2019) 14:e0208216. doi: 10.1371/journal.pone.0208216 30794556 PMC6386317

[50] Muenzer JT Davis CG Dunne BS Unsinger J Dunne WM Hotchkiss RS. Pneumonia after cecal ligation and puncture: A clinically relevant “two-hit” model of sepsis. Shock. (2006) 26:565–70. doi: 10.1097/01.shk.0000235130.82363.ed 17117130

