# Supplementary material for: Topical adjunctive treatment with flagellin augments pulmonary neutrophil responses and reduces bacterial dissemination in multidrug-resistant K. pneumoniae infection
Source: Front Immunol. 2024 Sep 4;15:1450486. doi: 10.3389/fimmu.2024.1450486 (PMC11408203; doi:10.3389/fimmu.2024.1450486)
Supplement: Supplementary file 1 [file DataSheet1.pdf]

Topical adjunctive treatment with flagellin augments pulmonary neutrophil responses and reduces bacterial dissemination in multidrug-resistant *Klebsiella* pneumonia

Christine C.A. van Linge[1,2], Robert F.J. Kullberg[1,2], Osoul Chouchane[1,2], Joris J.T.H. Roelofs[2,3], Wil H. F. Goessens[4], Cornelis van 't Veer[1,2], Jean-Claude Sirard[5], Alex F. de Vos[1,2], Tom van der Poll[1,2,6]

[1]Center for Experimental and Molecular Medicine, Amsterdam University Medical Center, Academic Medical Center, University of Amsterdam, Amsterdam, the Netherlands.
[2]Amsterdam Infection & Immunity Institute, Amsterdam, The Netherlands.
[3]Department of Pathology, Amsterdam University Medical Center, Academic Medical Center, University of Amsterdam, Amsterdam, The Netherlands.
[4]Department of Medical Microbiology and Infectious Diseases, Erasmus University Medical Center, Rotterdam, the Netherlands
[5]Center for Infection and Immunity of Lille, Institut Pasteur de Lille, U1019 - UMR9017, CHU Lille, CNRS, INSERM, University of Lille, Lille, France.
[6]Division of Infectious Diseases, Amsterdam University Medical Center, University of Amsterdam, Amsterdam, the Netherlands.

*** Correspondence:**
Christine van Linge, M.D.
c.c.vanlinge@amsterdamumc.nl

Supplementary material

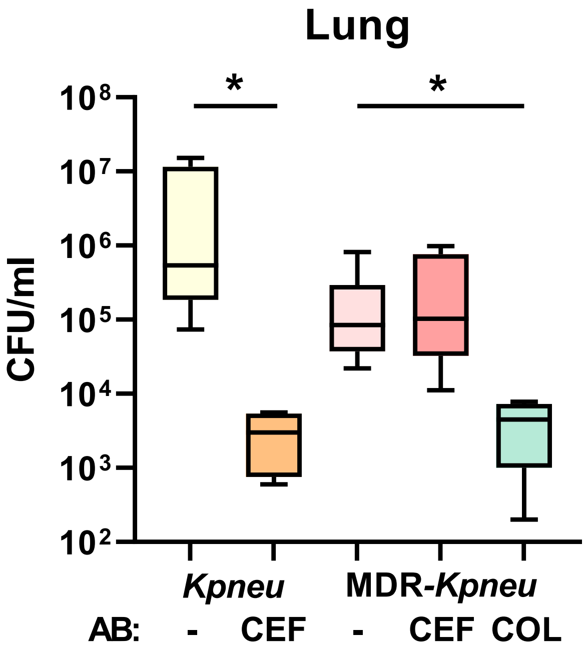

Fig. S1 Antibiotic susceptibility of *Kpneu* and MDR-*Kpneu in vivo*
Mice were infected with *Kpneu* or MDR-*Kpneu*, treated with ceftriaxone (cef), colistin (col) or placebo (-) after 6 hours, and sacrificed after 24 hours to determine CFU counts in the lung. Box and whiskers representing 4-8 mice per group. Differences were analysed using Mann-Whitney test. *P<0.05. Abbreviations: AB, antibiotics; CEF, ceftriaxone; COL, colistin; CFU, Colony Forming Units

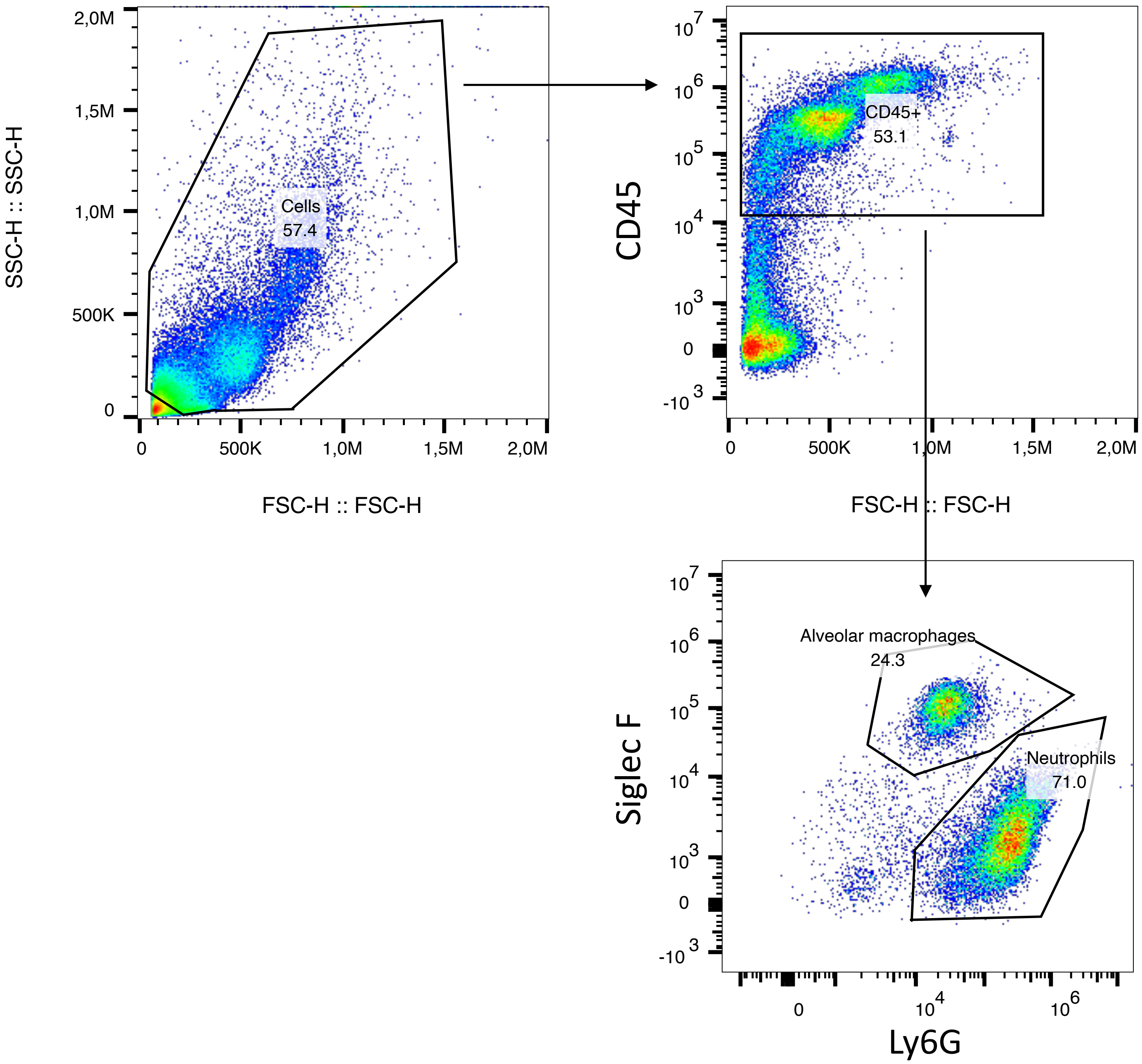

Fig. S2 Gating strategy of flow cytometry data to determine percentage of cell subsets in BALF

Gating strategy to determine percentages of alveolar macrophages ($CD45^{+}SigecF^{+}$) and neutrophils ($CD45^{+}Ly6G^{+}$).

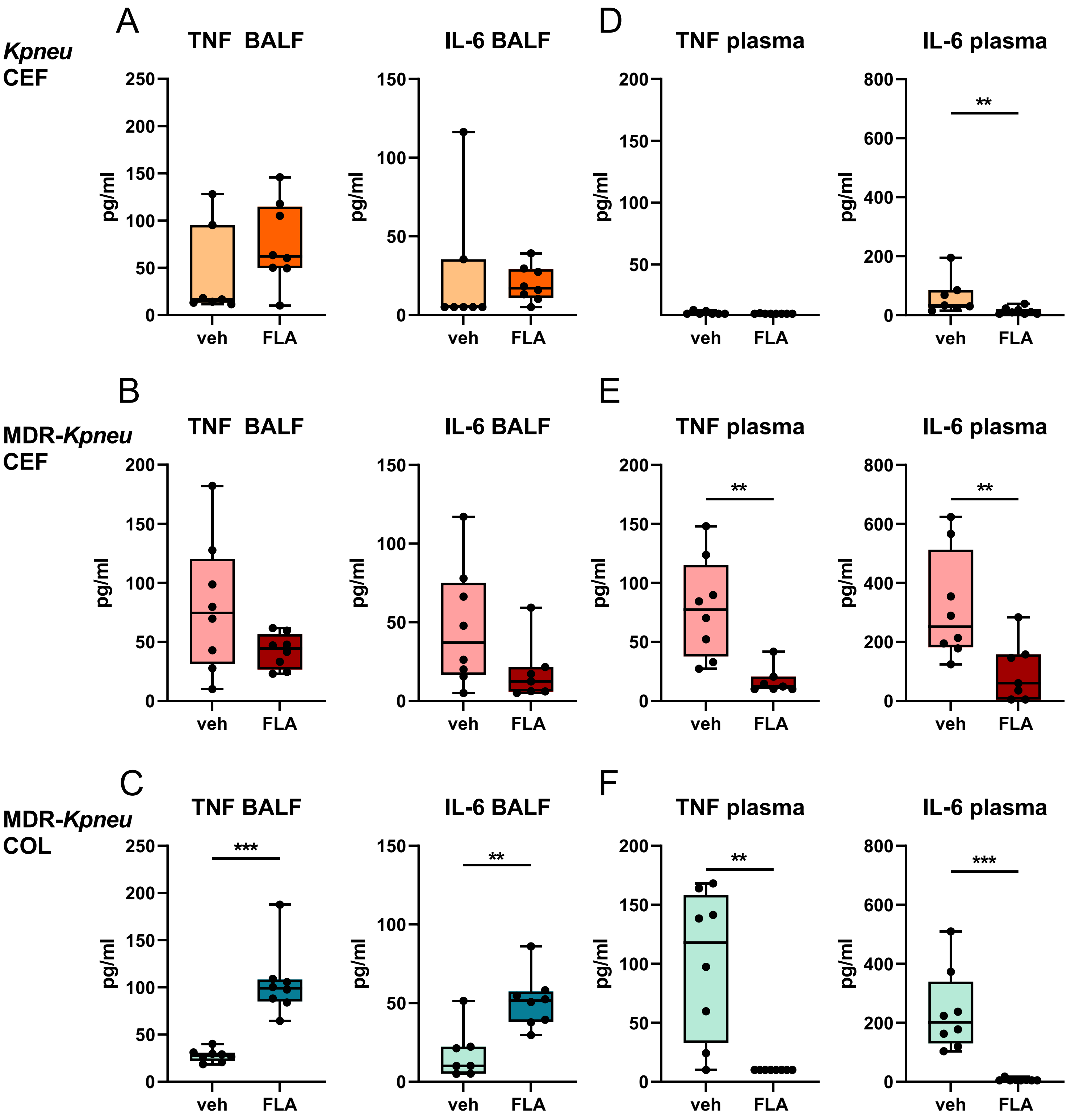

Fig. S3 Cytokine levels are higher in BALF and lower in plasma after flagellin treatment during antibiotic-treated MDR-*Kpneu* infection

Mice were infected with *Kpneu* and treated with ceftriaxone and vehicle or flagellin (A,D). Alternatively, mice were infected with MDR-*Kpneu* and treated with ceftriaxone and vehicle or flagellin (B,E), or with colistin and vehicle or flagellin after 6 hours (C,F). Endpoint of infection was after 24 hours. Cytokine concentrations of TNF and IL-6 in BALF (A-C) and in plasma (E-F). Box and whiskers representing 8 mice per group. Differences were analyzed using Mann-Whitney. **P<0.01, ***P<0.001. Abbreviations: veh, vehicle; FLA, flagellin; CEF, ceftriaxone; COL, colistin

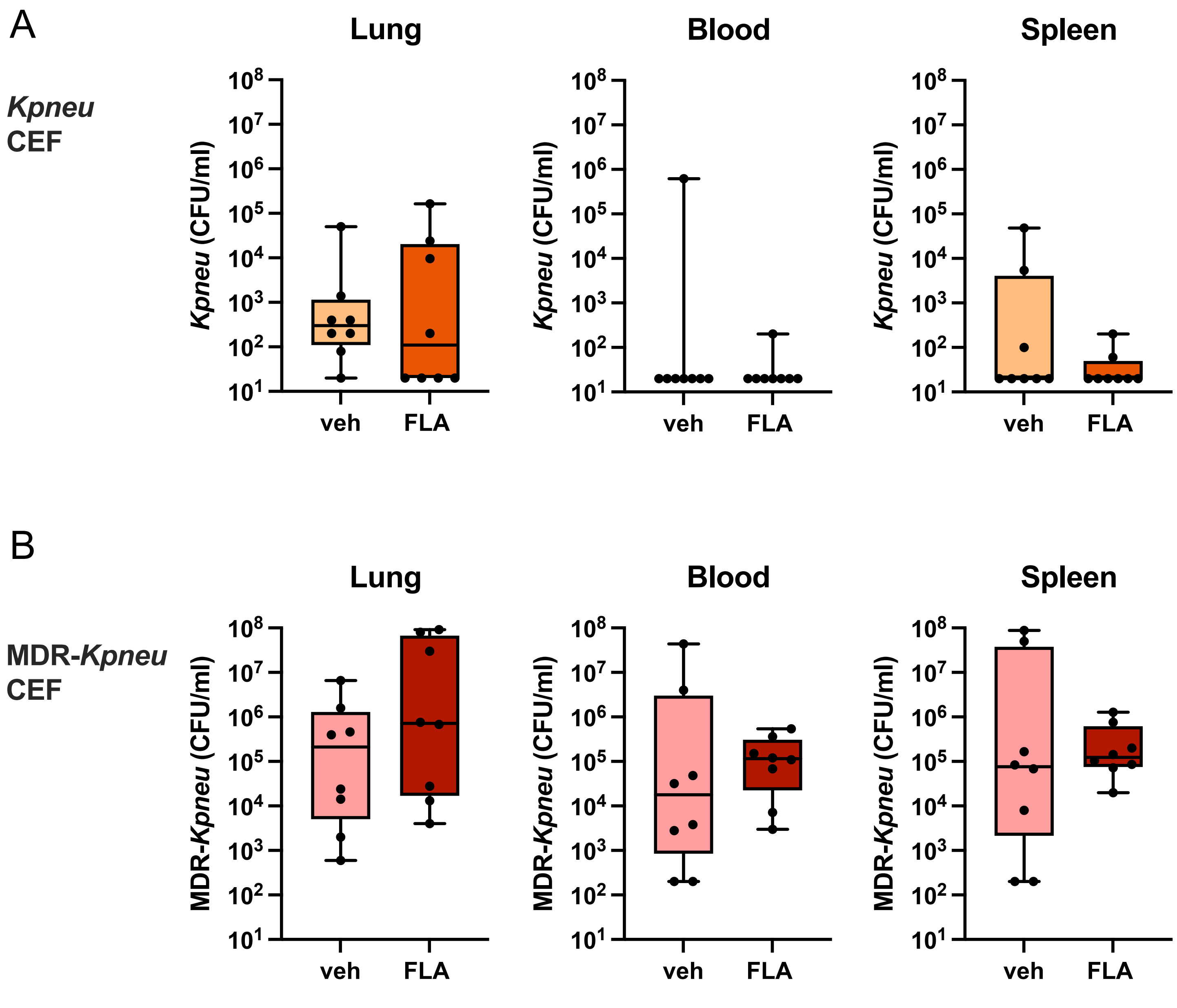

Fig. S4 Bacterial counts are not impacted by flagellin after 44 hours of infection
Mice were infected with *Kpneu* (A) or MDR-*Kpneu* (B) and treated with ceftriaxone and vehicle or flagellin after 6 and 28 hours. CFU counts in lung, blood and spleen were determined after 44 hours of infection. Box and whiskers representing 8 mice per group. Differences were analyzed using Mann-Whitney. $^{**}P<0.01$, $^{***}P<0.001$. Abbreviations: veh, vehicle; FLA, flagellin; CEF, ceftriaxone.

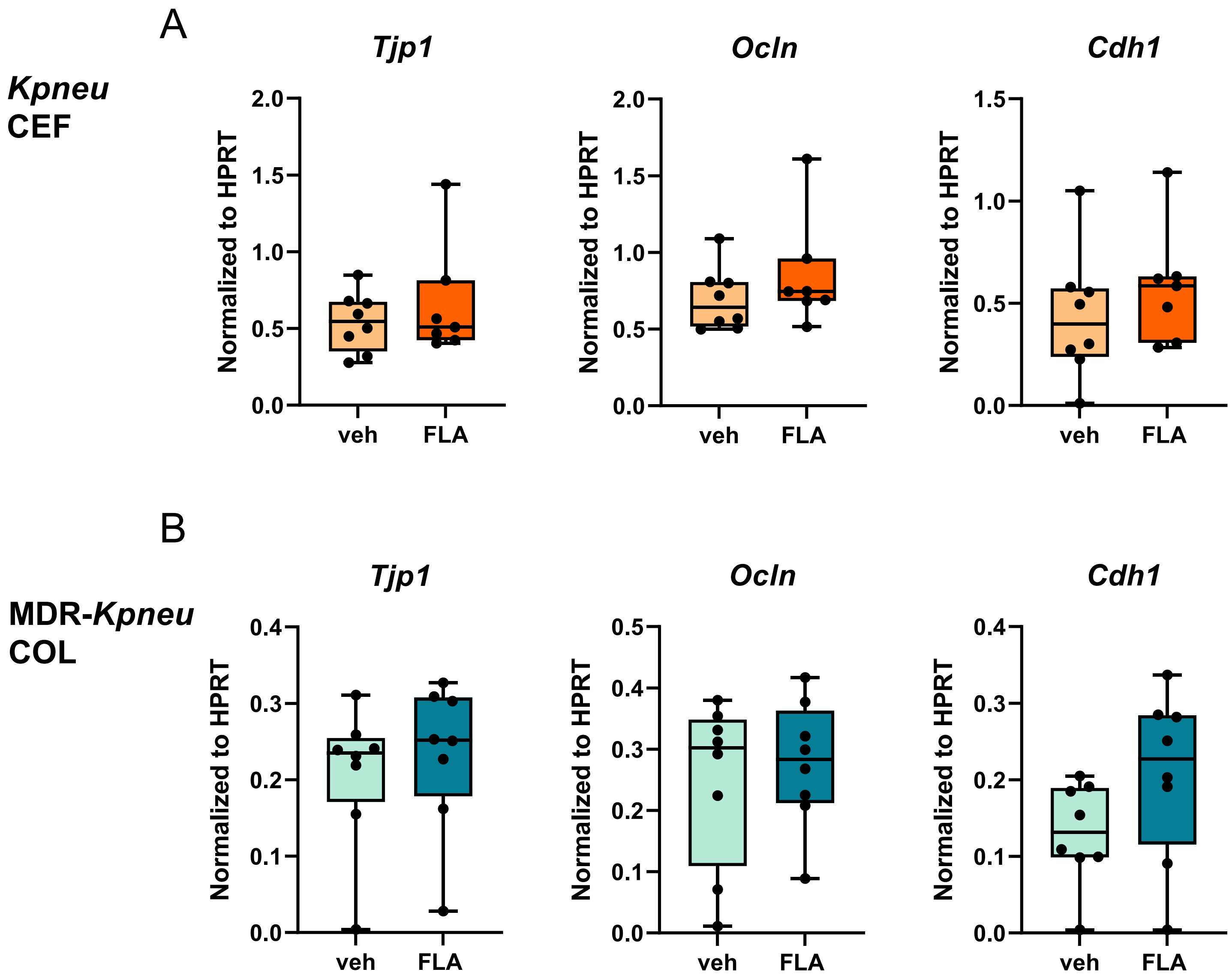

Fig. S5 Flagellin does not impact gene expression related to lung barrier function after 24 hours of antibiotically treated *Klebsiella* infection

Mice were infected with *Kpneu* and treated with ceftriaxone and vehicle or flagellin after 6 hours (A). Alternatively, mice were infected with MDR-*Kpneu* and treated with colistin and vehicle or flagellin after 6 hours. (B). Endpoint of infection was after 24 hours. mRNA expression normalized to HPRT in bronchial cells of epithelial integrity genes *Tjp1*, *Ocln*, and *Cdh1*. Box and whiskers representing 8 mice per group. Differences were analyzed using Mann-Whitney. Abbreviations: veh, vehicle; FLA, flagellin; CEF, ceftriaxone; COL, colistin

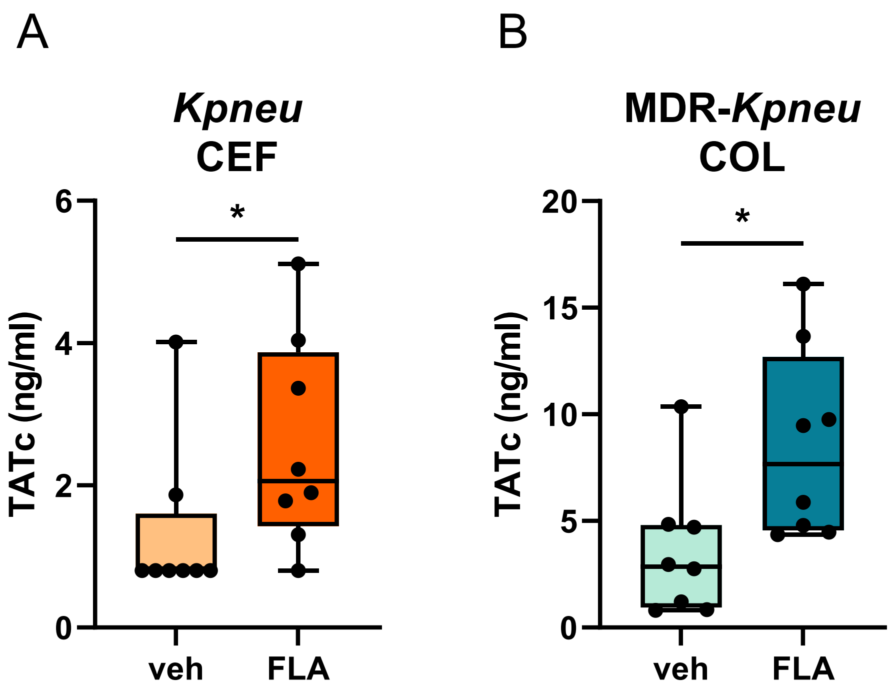

Fig. S6 Flagellin increases thrombin levels in BALF
Mice were infected with *Kpneu* and treated with ceftriaxone and vehicle or flagellin after 6 hours (A). Alternatively, mice were infected with MDR-*Kpneu* and treated with colistin and vehicle or flagellin after 6 hours. (B). Endpoint of infection was after 24 hours. TATc levels in BALF. Box and whiskers representing 8 mice per group. Differences were analyzed using Mann-Whitney test. *P<0.05, ***P<0.001. Abbreviations: veh, vehicle; FLA, flagellin; CEF, ceftriaxone; COL, colistin.

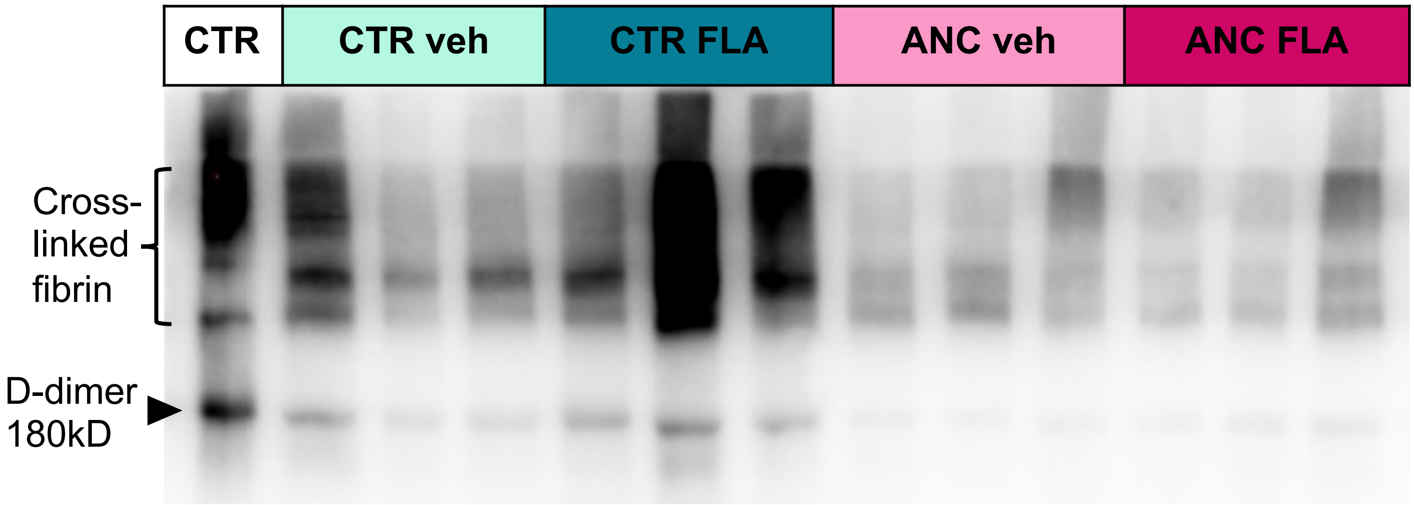

Fig. S7 Depletion of lung D-dimer and cross-linked fibrin in ancrod-treated mice
Mice were pre-treated with ancrod or saline as control 24 hours and immediately before infection with MDR-*Kpneu*. Subsequent treatment with colistin and flagellin or vehicle was administered after 6 hours and mice were sacrificed after 24 hours. Western blot of D-dimer (180kD) and cross-linked fibrin in lung homogenates, representative of three separate blots, in total 8 mice per group. Abbreviations: veh, vehicle; FLA, flagellin; CTR, control; ANC, ancrod

A

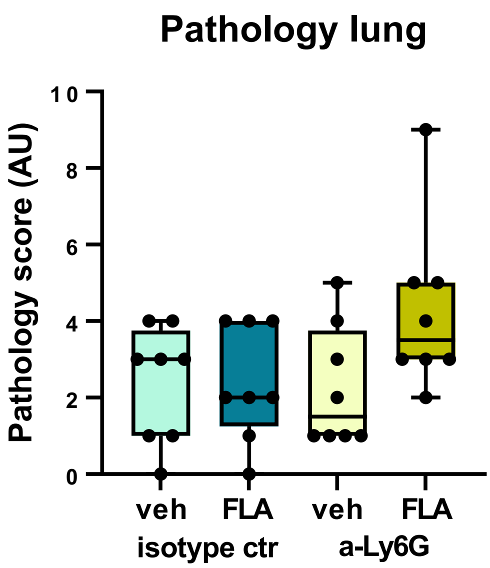

B

Bronchitis	veh CTR	FLA CTR	veh a-Ly6G	FLA a-Ly6G
Present	1*	5	0	1
Not present	7	3	8	7

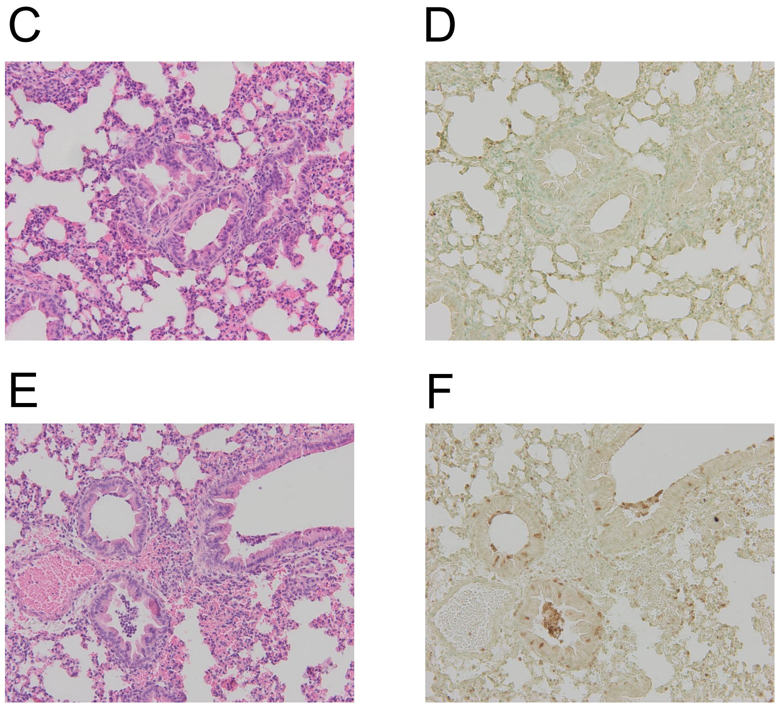

Fig. S8 Flagellin induces bronchial accumulation of neutrophils during antibiotic-treated *Klebsiella* infection

Mice were pre-treated with anti-Ly6G or isotype control 48, 24 and 3 hours before infection with MDR-*Kpneu*. In addition, all mice received anti-rat **κ** immunoglobulin light chain antibody as a secondary antibody 46 and 1 hour prior to bacterial inoculation. Treatment with colistin and flagellin or vehicle was administered after 6 hours and mice were sacrificed after 24 hours. Lung pathology scores (A), box and whiskers representing 8 mice per group. Presence of bronchitis per group (B). Representative photographs of Ly6G and H&E stained lung sections of vehicle- (C, D) and flagellin-treated (E, F) mice. Differences between groups were analyzed using Kruskall-Wallis (A), or chi-square test (B). Abbreviations: veh, vehicle; FLA, flagellin; CTR, isotype ctr.

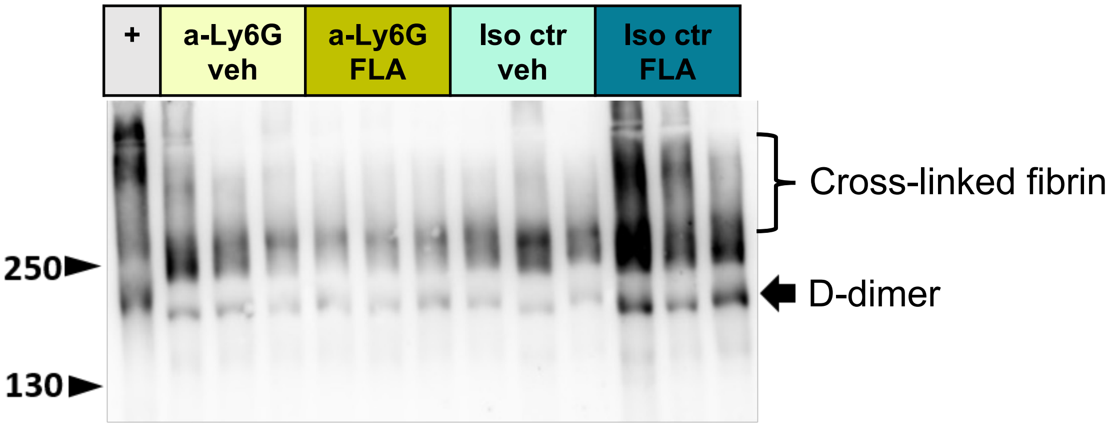

Fig. S9 Lung D-dimer and cross-linked fibrin in (PMN depleted-) mice
Mice were pre-treated with anti-Ly6G or isotype control 48, 24 and 3 hours before infection with MDR-*Kpneu*. In addition, all mice received anti-rat **κ** immunoglobulin light chain antibody as a secondary antibody 46 and 1 hour prior to bacterial inoculation. Subsequent treatment with colistin and flagellin or vehicle was administered after 6 hours and mice were sacrificed after 24 hours. Western blot of cross-linked fibrin and D-dimer, representative of three blots, in total 8 mice per group. Abbreviations: veh, vehicle; FLA, flagellin.

Gene	Forward	Reverse
Hprt	AGTCAAGGGCATATCCAACA	CAAACTTTGCTTTCCGGGT
Tjp1	ACTCCCACTTCCCCAAAAAC	CCACAGCTGAAG GACTCACA
Ocln	ACTGGGTCAGGGAATATCCA	TCAGCAGCAGCCATGTACTC
Cdh1	GCTCTCATCATCGCCACAGA	TTCGAGGTTCTGGGATGGGA

Table. S1 Primers used for qPCR
